# Talin–tensin3 interactions regulate fibrillar adhesion formation and tensin3 phase separation

**DOI:** 10.1083/jcb.202503155

**Published:** 2025-11-21

**Authors:** Xingchen Li, Rafaella Konstantinou, Vinod Kumar Meena, Saba Notash, Komal Khalil, Tom Whalley, Paul Atherton, Igor Barsukov, Thomas Zacharchenko, Christoph Ballestrem

**Affiliations:** 1 Faculty of Biology, Medicine and Health, Wellcome Centre for Cell-Matrix Research, University of Manchester, Manchester, UK; 2 Faculty of Health and Life Sciences, Institute of Systems, Molecular and Integrative Biology, University of Liverpool, Liverpool, UK

## Abstract

Integrin-mediated cell–matrix adhesions regulate communication between cells and the extracellular matrix. In matrix-secreting cells, fibrillar adhesions (FBs) containing high levels of α5β1 integrins and the tensin3 adaptor protein are essential for fibronectin (FN) fibrillogenesis. Here, we demonstrate that tensin3 binds to four helical regions (R3, R4, R8, and R11) of talin, the principal integrin activator. Structural analysis revealed the residues critical for the tensin3–talin interaction, and mutational analysis showed that talin R8 and R11 are essential for FB formation and FN fibrillogenesis. Cellular experiments demonstrate that tensin3 binding to talin not only regulates integrin activation, but also modulates tensin3’s propensity to undergo liquid–liquid phase separation (LLPS). Formation of such LLPS condensates increased when cells were plated on soft substrates compared with stiff ones. This effect was abolished by blocking the interaction between tensin3 and talin. Our data suggest a model in which LLPS condensates provide a signaling platform involved in cellular responses to sudden changes in tissue mechanics.

## Teaser

Li et al. show that multidomain interactions between talin and tensin3 regulate integrin activity, fibrillar adhesion formation, fibronectin assembly, and mechanosensitive tensin3 phase separation. Their findings shed light on a mechanism by which cells sense tissue mechanics and adapt adhesion signaling through the talin–tensin3 interaction.

## Graphical Abstract

**Figure:**
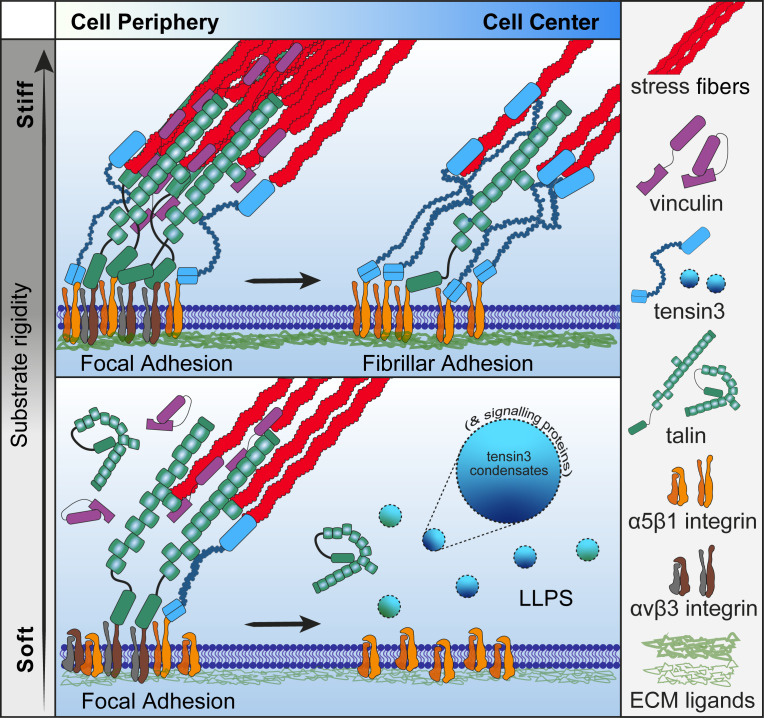

## Introduction

Cells are surrounded by the extracellular matrix (ECM), which provides essential mechanical support for tissue organization (Hynes, 2009). During development and disease, tissues can undergo dramatic changes in their biomechanical and chemical composition (Humphrey et al., 2014). Cells sense and respond to these changes in part by remodeling their matrix environment, but how this is regulated is not fully elucidated. Cell–matrix interactions are mediated by two closely related types of adhesion complexes, focal adhesions (FAs) and fibrillar adhesions (FBs) (Doyle et al., 2022; Wehrle-Haller, 2012). FAs provide traction forces at the cell periphery that are critical for cell migration (Balaban et al., 2001), whereas FBs develop from FAs and are involved in the assembly of FN fibrils in the central part of the cell (Pankov et al., 2000; Zamir et al., 2000).

Adhesion to the ECM in both FAs and FBs is mediated by integrin transmembrane receptors (Chastney et al., 2024). In FAs, integrins are maintained in an active, matrix-binding configuration by association of their cytoplasmic domains with several regulatory proteins, including the mechanosensory adaptor protein talin (Calderwood et al., 2013). Talin, together with another mechanosensor vinculin, couples FAs to actomyosin (Carisey and Ballestrem, 2011; Goult et al., 2021), which appears to be a prerequisite for FB formation and FN assembly (Lu et al., 2020; Zamir et al., 2000). In contrast to FAs, talin is almost absent in FBs (Katz et al., 2000), whereas tensin3, a member of the tensin family, is significantly enriched (Clark et al., 2010). Our previous work shows that tensin3 binding to talin is critical for the development of FAs into FBs (Atherton et al., 2022). However, the precise mechanism behind the functional transition from FAs to FBs, including changes in molecular composition, remains elusive (Geiger et al., 2001; Zamir et al., 1999).

Strikingly, unlike FAs, FBs persist when cells are treated with inhibitors that release actomyosin tension (Atherton et al., 2022). This persistence in a “force-free environment” requires integrins to be locked into an active ligand-bound conformation. While published data suggest that tensins play a role in this scenario (Atherton et al., 2022; Georgiadou et al., 2017; Rainero et al., 2015), the mechanism by which the intracellular molecular complexes in FBs remain stably associated with activated integrins is not understood.

**Figure 1.**
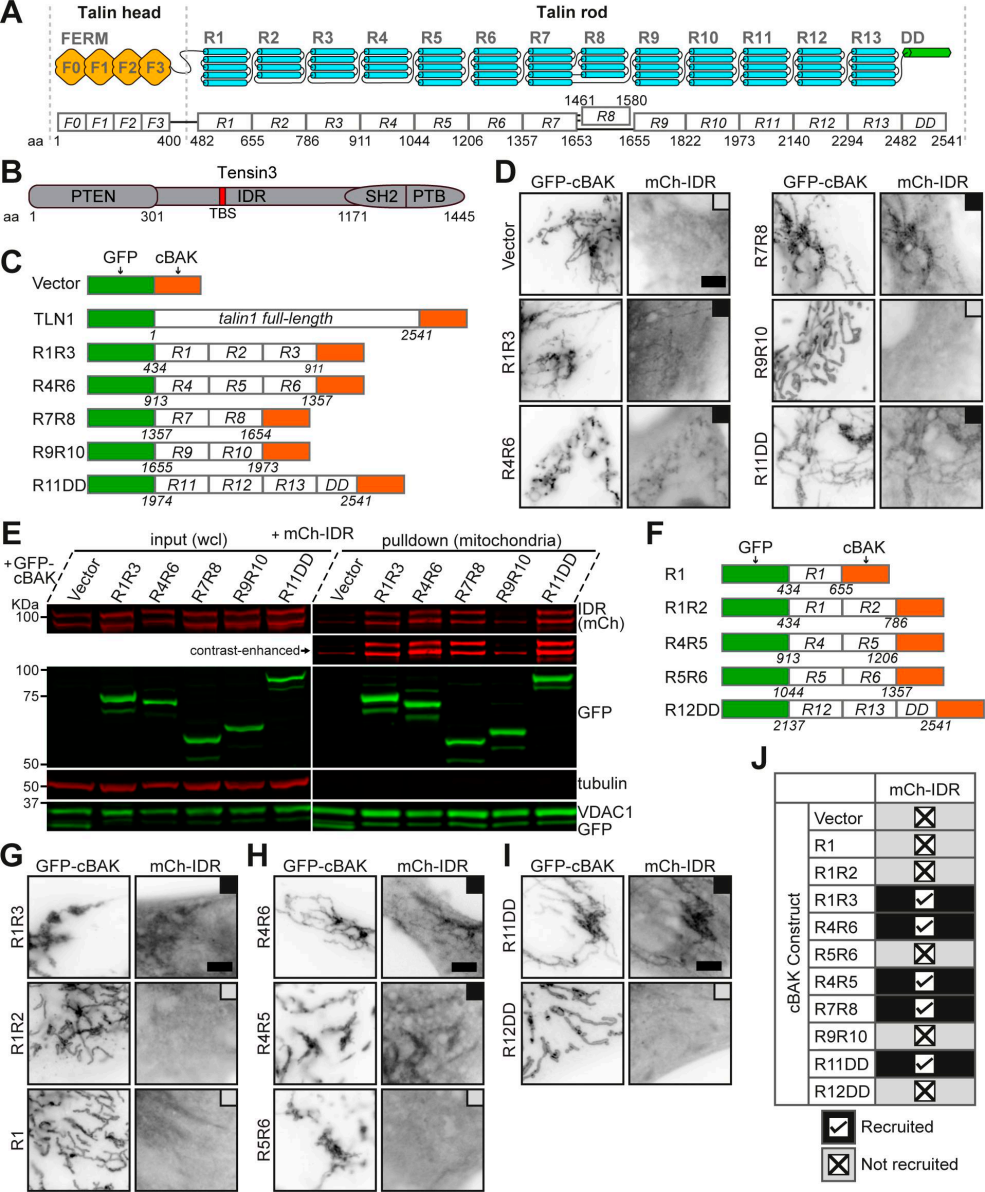
**Talin contains tensin-binding sites in the R3, R4, R7R8, and R11 domain. (A)** Schematic representation of talin with the aa numbers of each domain indicated. **(B)** Schematic representation of tensin3. Talin-binding site (TBS), aa 692–718. **(C)** Mitochondrial targeting talin1 full-length and deletion constructs. The indicated talin sequences were inserted between EGFP (GFP) and cBAK, with the aa number of talin regions indicated below. **(D)** Constructs shown in C were expressed with mCh-IDR in NIH3T3 cells. Black boxes indicate colocalization, and gray boxes indicate no association. **(E)** Western blotting of mitochondrial pulldown experiments. Constructs as used in D were expressed in HEK293T cells. Whole cell lysates (wcl) and purified mitochondria were immunoblotted. Note that the double band for mCh-IDR is due to known mCherry degradation. **(F)** Representation of additional talin1 constructs. **(G–I)** NIH3T3 cells expressing the constructs shown in C and F with mCh-IDR. **(J)** Summary table of the mitochondrial targeting assays. All results are collected from three independent experiments. Scale bars (D and G–I), 5 μm. aa, amino acid. Source data are available for this figure: SourceData F1.

The regulation of integrin activity is complex, and talin, which itself undergoes conformational changes and activation (Dedden et al., 2019; Goksoy et al., 2008), plays a central role in this process. Structurally, talin contains an N-terminal FERM domain (F0-F3) linked to 13 helical bundles (rod region, R1-R13) terminating in a dimerization domain (DD, Fig. 1 A). The FERM domain binds and activates integrins, while the rod region contains vinculin-binding sites and actin-binding sites that couple integrins to the actomyosin machinery (Calderwood et al., 2013; Goult et al., 2021). Tensin3, like talin, also binds to integrin (Calderwood et al., 2003; McCleverty et al., 2007) and may contribute to its activation (Georgiadou et al., 2017; Torgler et al., 2004), but mechanistic insight is lacking.

Tensin3 consists of an N-terminal PTEN homology domain, a large intrinsically disordered region (IDR), and C-terminal SH2 and PTB domains (Fig. 1 B). The conserved N- and C-terminal domains mediate the association with most known interaction partners (Liao and Lo, 2021), including the integrin-binding PTB domain (Calderwood et al., 2003; McCleverty et al., 2007). An exception is a talin-binding site (TBS) in the IDR, which is critical for FB formation and FN fibrillogenesis (Atherton et al., 2022).

The aim of this study was to gain structural and mechanistic insights into the talin–tensin3 interaction and its role in the regulation of integrin activity. We identified four tensin3-binding sites on the talin rod, including R3, R4, R8, and R11. We determined the structure of the R11R12–tensin3 complex and characterized the interactions of R3, R4, and R8 with tensin3 *in vitro*. The structural insights allowed the design of point mutations to investigate the functional relationship between tensin3 and talin with respect to integrin activation, FB formation, and FN fibrillogenesis. We show that talin activation modulates tensin3 binding, with activated talin promoting tensin3 retention in cell–matrix adhesions. Critically, tensin3 undergoes liquid–liquid phase separation (LLPS) that is mechanosensitive and dependent on the stiffness of the ECM substrate encountered by cells. This mechanosensitive phase separation of tensin3 is inversely correlated with the ability of talin to bind to tensin3 and can act as a platform for other adhesion and signaling proteins.

## Results

### Talin1 contains multiple binding sites for tensin3

**Figure S1.**
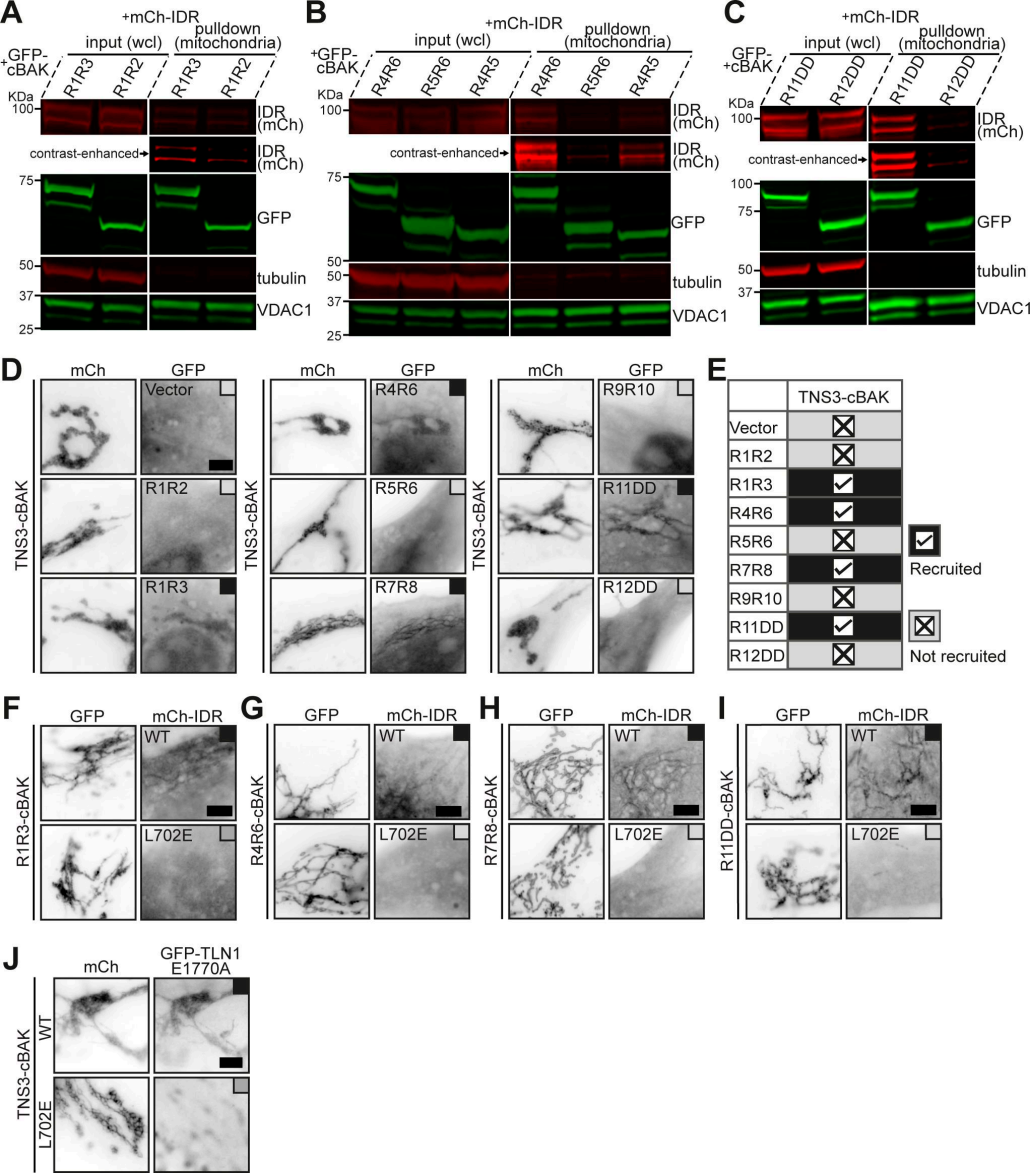
**Tensin3 binds multiple talin rod domains. (A–C)** Western blotting of the mitochondrial pulldown experiments in HEK293T cells with the same constructs used in Fig. 1, G–I, showing that R3, R4, and R11 interact with mCh-IDR. **(D)** Representative images of NIH3T3 cells co-expressing mCh-TNS3-cBAK with GFP-vector, GFP-R1R2, GFP-R1R3, GFP-R4R6, GFP-R5R6, GFP-R7R8, GFP-R9R10, GFP-R11DD, or GFP-R12DD, respectively. Black boxes indicate colocalization, and gray boxes indicate no association. Note that TNS3-cBAK recruits R1R3, R4R6, R7R8, and R11DD to the mitochondria, but not GFP-vector, R1R2, R5R6, R9R10, or R12DD. **(E)** Summary table of the MTS experiments in D. **(F–I)** Representative images of NIH3T3 cells co-expressing GFP-R1R3-cBAK (F), GFP-R4R6-cBAK (G), GFP-R7R8-cBAK (H), or GFP-R11DD-cBAK (I) with mCh-IDR-WT or mCh-IDR-L702E, respectively. Note that L702E abolishes mCh-IDR interactions with all talin1 truncation constructs. **(J)** Images of cells co-expressing mCh-TNS3-cBAK (WT or L702E) with GFP-TLN1-E1770A, respectively. L702E abolishes TNS3 colocalization with TLN1-E1770A at the mitochondria. All experiments are performed three times. Scale bars, 5 μm. Source data are available for this figure: SourceData FS1.

The talin rod consists of 13 domains comprised of bundles of four or five α-helices that share structural homology (Goult et al., 2013), and we have shown that talin R11 interacts with the tensin3 TBS (Atherton et al., 2022). However, the observations of dramatically increasing tensin3/talin ratios during the maturation from FAs to FBs suggested that the talin rod region might engage multiple tensins. To test this, we used a mitochondrial targeting system (MTS), involving fusion proteins to a mitochondrial targeting motif (cBAK) and co-expression of potential binding proteins (Atherton et al., 2020; Atherton et al., 2022; Li et al., 2023). In these assays using a series of structure-based talin1 rod deletion constructs (Fig. 1 C), we identified the regions of R1R3 (R1-R3), R4R6 (R4-R6), R7R8 and R11DD (R11-DD) as binding regions for tensin3 (Fig. 1 D), whereas R9R10 showed no interaction. These results were confirmed by mitochondrial pulldown experiments where all four talin regions coprecipitated with the tensin3 IDR in the isolated mitochondria from HEK293T cells (Fig. 1 E). The subsequent use of further talin deletion constructs (Fig. 1 F) demonstrated that the talin–tensin3 interaction involves talin R3, R4, R7R8, and R11 domains (Fig. 1, G–J and Fig. S1, A–E).

### Structure-based mutations in tensin3 disrupt its interaction with talin

**Figure 2.**
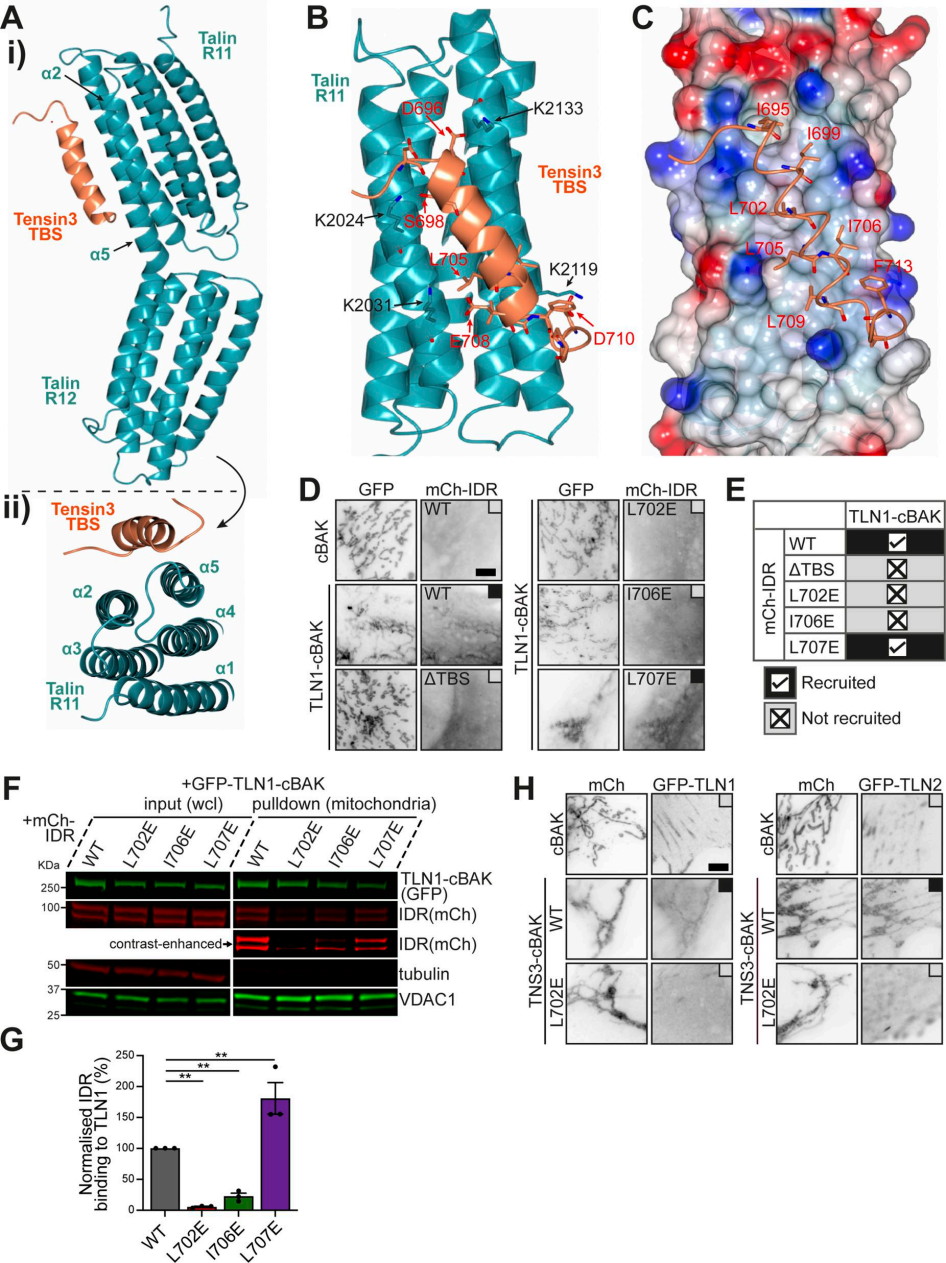
**Structure-based mutations in tensin3 disrupt its interaction with talin. (A)** Crystal structure of the talin R11R12–tensin3 TBS (aa 692–718) complex. **(i)** TBS (orange) forms a six-helix bundle with R11 (cyan). **(ii)** TBS engages the α2-α5 face of R11. **(B)** R11-TBS interface is stabilized by multiple electrostatic and hydrophobic interactions. K2024, K2119, and K2133 (black), form electrostatic contacts with D696, S698, and D710 (red). **(C)** Poisson–Boltzmann electrostatic distribution map of the tensin3-binding surface of R11. Tensin3 peptide is shown in sticky representation with the hydrophobic residues labeled (red). **(D)** GFP-TLN1-cBAK was co-expressed with mCh-IDR wild-type (WT), deletion of TBS (ΔTBS), or those carrying the point mutation L702E, I706E, or L707E, respectively, in NIH3T3 cells. Groups of GFP-cBAK and mCh-IDR-ΔTBS were used as negative controls. **(E)** Summary table of D. **(F)** Mitochondrial pulldown experiment with the constructs used in D. **(G)** Quantification of F from triplicate experiments. Data are normalized to WT. Error bars are SEM; ** indicates P < 0.01 (ordinary one-way ANOVA with Dunnett’s multiple comparisons). **(H)** Representative images of NIH3T3 cells expressing mCh-TNS3-WT-cBAK or mCh-TNS3-L702E-cBAK and GFP-TLN1 (left panel) or GFP-TLN2 (right panel). mCh-cBAK was used as a negative control to recruit GFP-TLN1 and GFP-TLN2 to the mitochondria. Data are collected from three independent experiments. Scale bars (D and H), 5 μm. Source data are available for this figure: SourceData F2.

**Figure S2.**
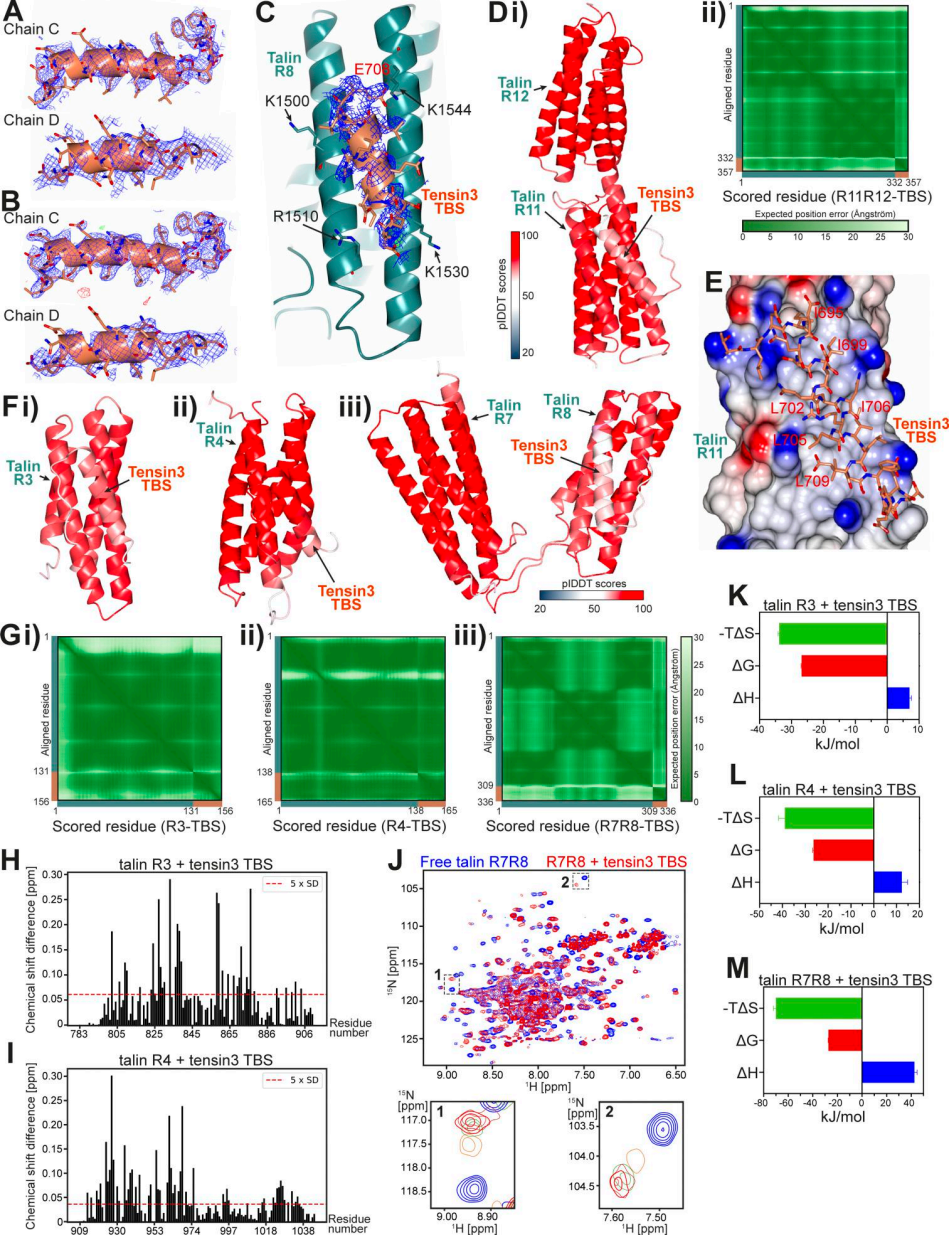
**Structural determination of the talin–tensin3 interaction. (A)** SA composite omit map of tensin3 TBS electron density focused on chains C and D and contoured at 1σ (blue). **(B)** Refined 2F_0_-F_C_ map of tensin3 TBS chain C and D contoured at 1σ (blue), with F_0_-F_C_ map contoured at 3.5σ (+ve, green/−ve, red). **(C)** Electron density of the talin R8-tensin3 TBS complex (∼2.6 Å), with the refined 2F_0_-F_C_ map of tensin3 TBS (orange) contoured at 0.7σ (blue). Talin R8, cyan. **(D i and ii)** AlphaFold3 model of the talin R11R12-tensin3 TBS complex (i) colored by confidence according to the pIDDT score with TBS facing forward, and contact prediction plot (ii) showing the expected positional error per residue in Angstrom (Å) ranging from 0 Å (dark green) to 30 Å (white). Note that the input talin R11R12 (cyan) and tensin3 TBS (orange) sequences are marked on the plot axes. **(E)** Poisson–Boltzmann electrostatic distribution map of the tensin3-binding surface of R11 in the predicted complex. Tensin3 peptide is shown in a stick representation with the hydrophobic residues labeled (red). The predicted R11-TBS complex is identical to the crystal structure shown in Fig. 2, A–C. **(F i–iii)** AlphaFold3 model of tensin3 TBS in complex with talin R3 (i), R4 (ii), and R7R8 (iii) with TBS facing forward. **(G i–iii)** Contact prediction plots related to F for the models of R3-TBS (i), R4-TBS (ii), and R7R8-TBS (iii). Note that the input talin (R3, R4, and R7R8; cyan) and tensin3 TBS (orange) sequences are marked on the plot axes. **(H and I)** Residue-specific CSD of talin R3 (H) and R4 (I) upon the addition of tensin3 TBS peptide at a 1:2 M ratio. The dashed line indicates the significant difference threshold of 5× SD. Mapping of CSD on the AlphaFold3 models is shown in Fig. 3, I and J. **(J)** Overlay ^1^H-^15^N HSQC spectra of ^15^N-labeled talin R7R8 (200 µM) in the absence (blue) and presence (red) of tensin3 TBS peptide at a 1:4 M ratio. Dashed boxes in the full spectra are magnified in the lower panels, illustrating the progressive chemical shift changes at peptide molar ratios of 0 (blue), 0.5 (orange), 1.0 (green), 2.0 (coral), and 4.0 (red). The HSQC spectra were recorded at 800 MHz. **(K)** Thermodynamic parameters of the talin R3–tensin3 TBS interaction. Red, blue, and green bars represent Gibbs free energy (ΔG = −26.8 ± 0.20 kJ/mol), enthalpy change (ΔH = 7.02 ± 0.62 kJ/mol), and entropy contribution (−TΔS = −33.8 ± 0.40 kJ/mol), respectively. **(L)** Thermodynamic parameters of the talin R4–tensin3 TBS interaction. ΔG = −26.3 ± 0.36 kJ/mol; ΔH = 12.3 ± 2.45 kJ/mol; -TΔS = −39.0 ± 2.62 kJ/mol. **(M)** Thermodynamic parameters of the talin R7R8–tensin3 TBS interaction. ΔG = −27.40 ± 0.17 kJ/mol; ΔH = 42.72 ± 2.06 kJ/mol; −TΔS = −70.10 ± 1.94 kJ/mol. Values in K‑M represent mean ± SD from triplicate measurements. CSD, chemical shift difference; SA, simulated annealing.

To further characterize talin interactions with tensin3, we aimed to gain structural insight into the talin–tensin3 associations. We first determined the structure of the talin–R11R12 in complex with tensin3 TBS to 2.76 Å resolution (Fig. 2 A and Table S1). This revealed that the tensin3 TBS forms an amphipathic α-helix that binds between the α2-α5 helices of the R11 bundle, forming a 6-helix bundle (Fig. 2 A; and Fig. S2, A and B). Residues D696, S698, and D710 on tensin3 TBS establish electrostatic contacts with K2024, K2119, and K2133 on R11 (Fig. 2 B), while K2031 orients its sidechain away from L705 on tensin3. The hydrophobic surface of R11 engages with the uncharged face of TBS, with tensin3 residues L702 and I706 forming the core of the hydrophobic interface (Fig. 2 C), demonstrating that these sites are critical for tensin3 interaction with talin R11. To test their importance, we introduced single negative charge mutations of L702E and I706E in the tensin3 IDR (mCh-IDR), which were then tested for talin binding in MTS experiments (Fig. 2 D). While GFP-TLN1-cBAK readily recruited mCh-IDR to the mitochondria, both L702E and I706E mutations completely abolished colocalization with talin (Fig. 2, D and E), as seen for deletion of the TBS (ΔTBS). A control mutation L707E did not affect the colocalization of mCh-IDR with GFP-TLN1-cBAK. Mitochondrial pulldown experiments confirmed these data (Fig. 2, F and G), showing that L702E completely abolished and I706E strongly reduced mCh-IDR binding to talin. Further experiments showed that L702E abolished mCh-IDR binding to all four talin regions (Fig. S1, F–I). Thus, L702 is critical for tensin3 interactions with all binding sites in talin, and these interactions share a similar mechanism. In addition, L702E disrupted tensin3 interactions with both talin1 and talin2 (GFP-TLN1 and GFP-TLN2, Fig. 2 H), and a constitutively active talin (GFP-TLN1-E1770A, Fig. S1 J), demonstrating that the disruption does not depend on the talin activation state.

### Structural insights reveal essential residues in talin R11 and R8 for tensin3 binding

**Figure 3.**
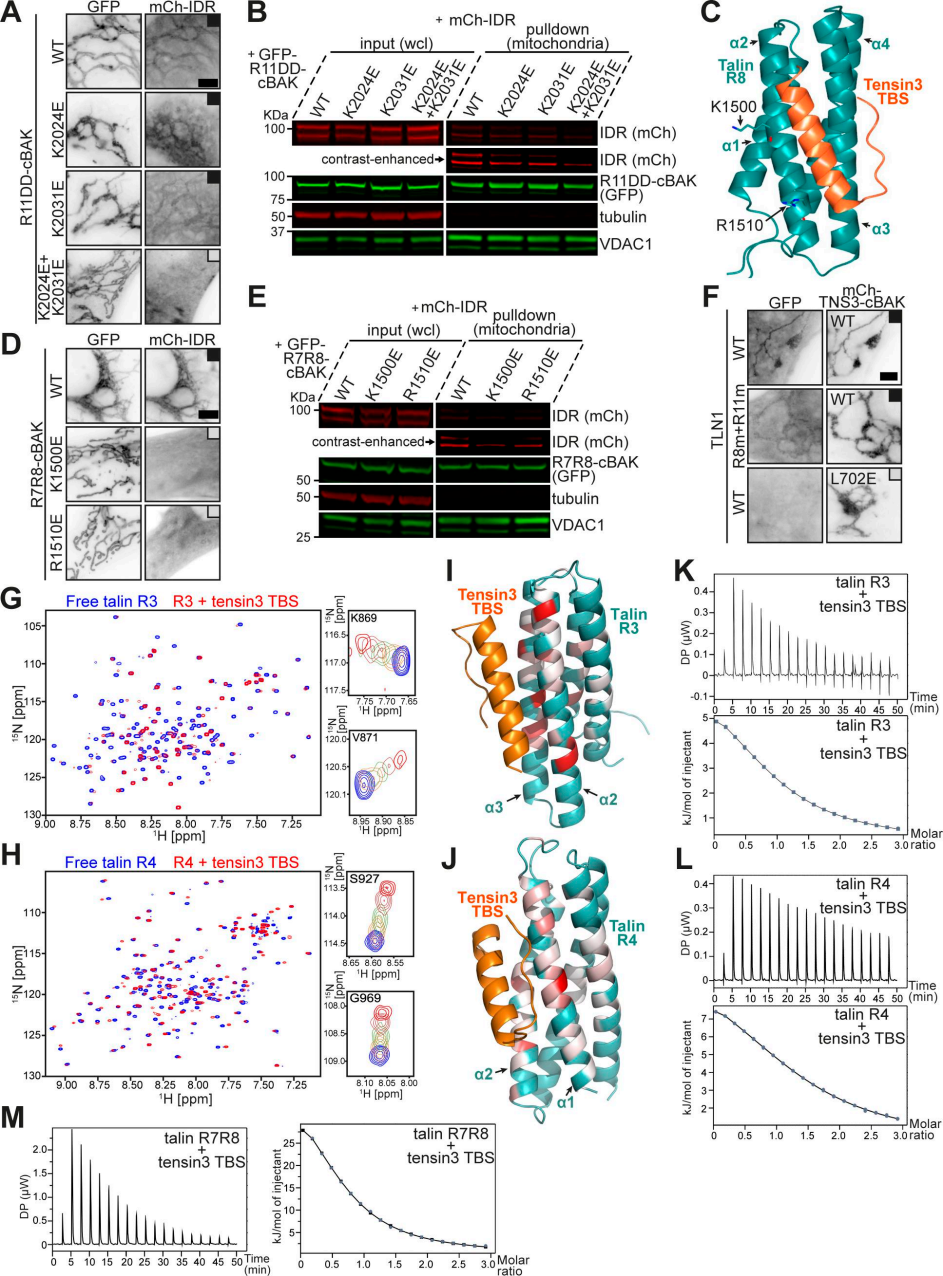
**Structural characterization of the multidomain talin–tensin3 interaction. (A)** Representative images of NIH3T3 cells co-expressing GFP-R11DD-cBAK WT or carrying K2024E, K2031E, or both (K2024E+K2031E) with mCh-IDR. **(B)** Mitochondrial pulldown experiment using the same constructs as in A. **(C)** AlphaFold3 model of the talin R8–tensin3 TBS complex. TBS (orange) was predicted to engage the α2-α3 face of the R8 bundle (cyan). K1500 and R1510 (brown) are highlighted on the R11 domain. **(D)** NIH3T3 cells co-expressing GFP-R7R8-cBAK WT or carrying K1500E or R1510E with mCh-IDR. **(E)** Mitochondrial pulldown experiment using constructs as in D. **(F)** Cells co-expressing mCh-TNS3-WT-cBAK and GFP-TLN1 constructs (R8m, K1500E; R11m, K2024E+K2031E). mCh-TNS3-L702E-cBAK was used as a negative control. **(G and H)** Overlay ^1^H-^15^N HSQC spectra of ^15^N-labeled talin R3 (G) and talin R4 (H), at a concentration of 200 µM, in the absence (blue) and presence (red) of tensin3 TBS peptide at a molar ratio of 1:2. Magnified views in the right panels show the cross-peaks corresponding to the residues K869 and V871 of talin R3 (G) and the residues S927 and G969 of talin R4 (H), illustrating the progressive chemical shift changes at peptide molar ratios of 0 (blue), 0.25 (orange), 0.5 (green), 1.0 (coral), and 2.0 (red). The HSQC spectra of R3 and R4 were recorded at 700 and 800 MHz, respectively. **(I and J)** Mapping of the residue-specific CSD (related to Fig. S2, H and I) on the AlphaFold3 models of talin R3 (I) and R4 (J) colored in cyan, respectively, in complex with tensin3 TBS (orange). Residues with significant CSDs are colored red, using a red-white linear gradient scale with red corresponding to the maximum CSD and white to the threshold. Residues with CSDs below the threshold are colored in green. Images are generated using PyMOL. **(K–M)** ITC profiles of the talin R3 (K), R4 (L), and R7R8 (M) interaction with tensin3 TBS, respectively. The upper panels (and left panel in M) show the raw heat flow data obtained during the titration of 600 µM of tensin3 TBS peptide into 40 µM talin R3 (K) or R7R8 (M), or the titration of 450 µM of tensin3 TBS peptide into 30 µM talin R4 (L), respectively, at a temperature of 25°C. The lower panels (and right panel in M) represent the integrated heat per injection plotted against the molar ratio. The data were fitted using a single-site binding model with dissociation constants (K_d_) of 20.5 ± 1.80 µM for the R3-TBS interaction (K), 23.8 ± 2.94 µM for the R4-TBS interaction (L), and 15.8 ± 0.90 µM for the R7R8-TBS interaction. All results are collected from three independent experiments. Scale bars (A, D, and F), 5 μm. CSD, chemical shift difference. Source data are available for this figure: SourceData F3.

The R11-TBS structure demonstrated that the positively charged residues K2024 and K2031 on talin are involved in R11 binding to tensin3 TBS (Fig. 2 B). To test this, these residues were replaced by glutamate and introduced into GFP-R11DD-cBAK and examined in our MTS assays. Results showed that single point mutations of either K2024E or K2031E only partially reduced the mitochondrial recruitment of mCh-IDR. However, the double mutation (K2024E+K2031E), referred to hereafter as the “R11m” mutant, completely abolished the recruitment (Fig. 3 A). Mitochondrial pulldown experiments confirmed these results (Fig. 3 B).

The tensin3 complexes of the other tensin3-binding domains that we detected in cell experiments either failed to crystallize (R3 and R4) or did not have sufficient quality of the electron density to accurately resolve the peptide sidechains (R7R8, ∼2.6 Å resolution, Fig. S2 C). We therefore modeled the complexes using AlphaFold3 and low-quality electron density, which supports the general location and orientation of the helix on R7R8. This approach is justified by comparing the predicted structure and our experimental structure of the R11R12–tensin3 TBS complex, which are almost identical (Fig. S2, D and E).

In agreement with our MTS assays (Fig. 1 and Fig. S1), AlphaFold3 predicted with high confidence that R3, R4, and R8 form complexes with tensin3 TBS (Fig. S2, F and G). Although the R7R8–tensin3 TBS complex had poor sidechain definition, we were able to confirm the binding site and polarity of the peptide (Fig. S2 C). These results were consistent with AlphaFold3 modeling (Fig. S2 F) and were very similar to the DLC1-R8 interaction (Zacharchenko et al., 2016). In the predicted structure, K1500 and R1510 of R8 are located at the interface with the tensin3 TBS (Fig. 3 C). Introducing K1500E and R1510E mutants in GFP-R7R8-cBAK abolished mCh-IDR recruitment to mitochondria (Fig. 3, D and E). Hereafter, we refer to the K1500E mutation as “R8m.” In the FL talin construct, the simultaneous introduction of R8m and R11m failed to abolish interaction with tensin3 (Fig. 3 F), suggesting that both R3 and R4 domains still contribute to the tensin3–talin interaction. However, mutation of a set of residues (listed in Table S2) failed to abolish talin R3 or R4 interaction with tensin3.

### Characterization of talin R3, R4, and R7R8 interaction with tensin3 TBS

To characterize the R3, R4, and R7R8 interactions in more detail, we conducted nuclear magnetic resonance (NMR) experiments with chemical shift mapping using recombinant talin R3 domain (residues 787–911), R4 domain (residues 913–1,044), and R7R8 region (residues 1,359–1,659) with tensin3 TBS. The addition of tensin3 TBS to the ^15^N-labeled talin R3 or R4 led to substantial changes in the respective ^1^H, ^15^N HSQC spectra (Fig. 3, G and H). Gradual chemical shifts and peak broadening were observed in a concentration-dependent manner, with significant perturbations seen in the α2 and α3 helices of R3 (Fig. S2 H and Fig. 3 I) and the α1 and α2 helices of R4 (Fig. S2 I and Fig. 3 J), which correlate with the AlphaFold3 models. We noticed minor changes in the other talin R3 and R4 helices, suggesting that TBS engagement affects the overall structure of talin R3 and R4. For talin R7R8, we observed similar concentration-dependent chemical changes upon the addition of tensin3 TBS (Fig. S2 J). Although we did not have resonance assignments to map the chemical shift changes on the structure, the observed electron density clearly indicates the location of the bound peptide.

The chemical shifts observed in the R3, R4, and R7R8 spectra suggest that the intermediate exchange rate for the tensin peptide is typically associated with a dissociation constant (K_d_) in the micromolar (µM) range. We then conducted isothermal titration calorimetry (ITC) experiments to measure the K_d_ and other thermodynamic parameters. Concentration-dependent heat absorption (positive peaks) was observed when the R3, R4, and R7R8 solutions were titrated with the TBS peptide (Fig. 3, K–M, respectively), indicating endothermic binding. The binding curves were fitted using a single-site binding model, yielding a K_d_ of 20.5 ± 1.8 μM for talin R3 with a positive enthalpy change of 7.02 ± 0.6 kJ/mol (Fig. 3 K and Fig. S2 K). The K_d_ for talin R4 is slightly higher (23.8 ± 2.9 μM) with an enthalpy of 12.3 ± 2.4 kJ/mol (Fig. 3 L and Fig. S2 L). Talin R7R8 is characterized with the lowest K_d_ (15.8 ± 0.9 μM), with an enthalpy of 42.72 ± 2.06 kJ/mol. These endothermic binding profiles are similar to our previous observation regarding talin R11 with tensin3 TBS (17 μM) (Atherton et al., 2022).

Together, these results confirmed our MTS data and AlphaFold3 prediction that talin R3, R4, and R7R8 are bona fide binding domains for tensin3 TBS. While the binding of tensin3 TBS is mediated by the α2 and α3 helices of R3 and the α1 and α2 helices of R4, further investigation is needed to identify the key residues in talin R3 and R4 that are responsible for tensin3 binding.

### The tensin3 talin binding–deficient mutant L702E blocks FB formation and fibronectin fibrillogenesis

**Figure 4.**
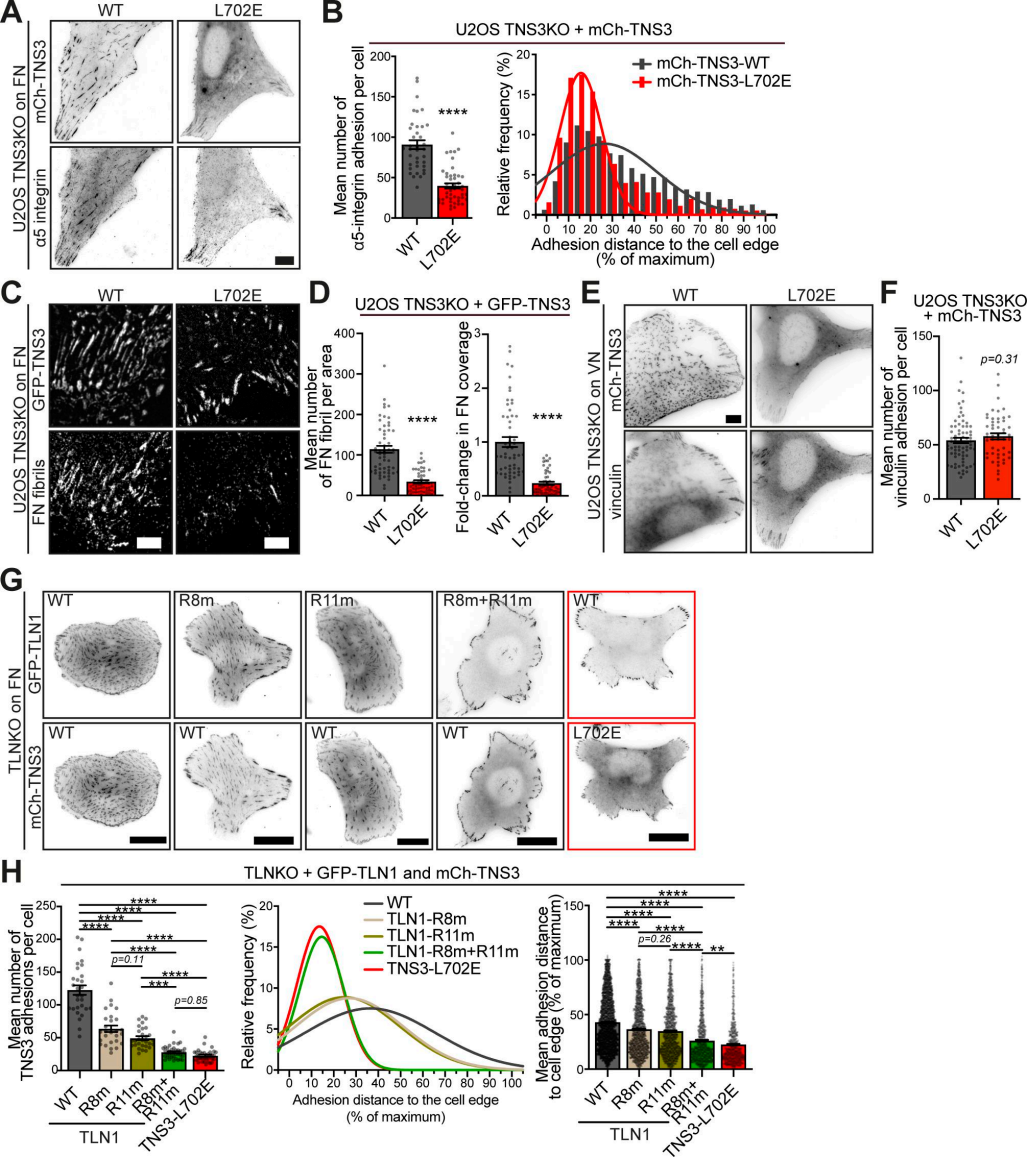
**Tensin3–talin interaction controls FB formation and FN fibrillogenesis. (A)** Representative images of U2OS TNS3KO cells expressing mCh-TNS3-WT or -L702E spread overnight on FN-coated glass-bottom dishes, before being stained for α5 integrins (SNAKA51). **(B)** Left panel: Quantification of the mean number of α5 integrin-positive adhesions in A; *n* = 37 (WT) and 47 (L702E) cells. Right panel: Histograms and associated Gaussian curve fits for the frequency of the normalized distance (percentage of maximum) to the cell edge of each α5 integrin–positive adhesion; *n* = 3,335 (WT, 37 cells) and 1,860 (L702E, 47 cells) adhesions, respectively. **(C)** Images (background-subtracted) of cell-derived FN fibrils (labeled with IST9 antibody) produced by TNS3KO cells expressing GFP-TNS3-WT or GFP-TNS3-L702E, spread overnight on FN-coated glass. **(D)** Left panel: Quantification of FN fibrils in 40 × 40 μm square areas applied to each image; *n* = 56 (WT) and 54 (L702E) cells. Right panel: Fold change in FN-covered area (normalized to WT). **(E)** Images of U2OS TNS3KO cells expressing mCh-TNS3-WT or mCh-TNS3-L702E spread overnight in serum-free medium on VN-coated glass-bottom dishes, before being fixed and stained for the FA marker vinculin. **(F)** Quantification of the mean number of vinculin-positive adhesions in E; *n* = 74 (WT) and 51 (L702E) cells. **(G)** Images of TLNKO cells co-expressing GFP-TLN1 constructs (WT, R8m, R11m, or R8m+R11m) with mCh-TNS3-WT. Cells co-expressing GFP-TLN1-WT and mCh-TNS3-L702E (in the red box) were used as a negative control as the complete disruption of the talin–tensin3 interaction. **(H)** Left panel: Quantification of TNS3-positive adhesions in G. *n* = 29 (WT), 27 (R8m), 29 (R11m), 37 (R8m+R11m), and 35 (L702E) cells. Middle and right panels: Gaussian curve fits and means for the normalized distance (percentage of maximum) to the cell edge of each TNS3-positive adhesion in G; *n* = 3,528 (WT), 1,720 (R8m), 1,433 (R11m), 1,030 (R8m+R11m), and 790 (L702E) adhesions from 29, 27, 29, 37, and 35 cells, respectively. ** indicates P < 0.01; *** indicates P < 0.001; **** indicates P < 0.0001 (B and D: Mann–Whitney test; F: unpaired *t* test; H left panel: Ordinary one-way ANOVA test with Tukey’s multiple comparisons test; H right panel: Kruskal–Wallis test with Dunn’s multiple comparisons test). All results are collected from three independent experiments. Scale bars: 10 μm (A and E), 20 μm (G), and 5 μm (C). Error bars are the SEM.

Tensin plays a critical role in the formation of α5β1-enriched FBs, which are frequently associated with FN fibrillogenesis (Pankov et al., 2000). We have previously shown that tensin3 knockout (TNS3KO) cells are largely deficient in these FBs (Atherton et al., 2022). To evaluate the impact of L702E on FB formation, we performed rescue experiments in TNS3KO cells. These cells were plated on FN and stained for α5 integrins (Fig. 4 A). Cells expressing mCh-TNS3-WT showed α5 integrin-positive adhesions in the peripheral and central areas of the cells (Fig. 4 B). In contrast, cells expressing mCh-TNS3-L702E showed a 55% reduction in centrally located α5 integrin-positive FBs. To examine the effect of L702E on FB-associated FN fibrils, we stained the rescued cells with an antibody that labels cellular FN (Fig. 4 C). Quantification shows that cells expressing TNS3-L702E produced over 70% fewer FN fibrils compared with cells expressing TNS3-WT (Fig. 4 D).

While α5β1 integrins are critical for FB formation, αvβ3 integrins, whose main ligand is vitronectin (VN), are enriched in FAs (Ballestrem et al., 2001; Zamir et al., 2000). To assess whether the talin–tensin3 interaction affects FA formation, we plated the cells on VN and stained for vinculin (Fig. 4, E and F). The results showed that there was no difference in the formation of vinculin-positive FA between U2OS TNS3KO cells expressing TNS3-WT and TNS3-L702E.

Together, these results demonstrate that the single L702E mutation in tensin3 abolishes FB formation and FN fibrillogenesis, but does not affect FA formation.

### Talin R8 and R11 are the critical functional interaction sites for tensin3-mediated FB formation

To investigate the functional role of specific tensin3 interaction sites located in the talin rod, we performed rescue experiments in cells lacking talin1 and talin 2 double knockout (TLNKO) cells (Atherton et al., 2015). In these experiments, we co-expressed WT or mutant GFP-TLN1 constructs together with mCh-TNS3 constructs in TLNKO cells (Fig. 4 G). In contrast to control cells co-expressing WT forms of talin1 and tensin3, the co-expression of TNS3-WT with TLN1-R8m or TLN1-R11m reduced the number of adhesions by ∼48% and 59%, respectively (Fig. 4 H); both mutants had fewer tensin3-positive adhesions particularly in the center of the cells. Notably, the expression of the TLN1-R8m+R11m double mutant resulted in the greatest reduction in tensin3-positive adhesions (∼77% compared with WT, Fig. 4 H), with centrally located adhesions being almost absent, similar to that observed in cells expressing GFP-TLN1-WT with mCh-TNS3-L702E. These data suggest that tensin3 binding to the talin R8 and R11 domains is critical for the efficient formation of FBs.

### Tensin3 regulates integrin activation through its interaction with talin

**Figure S3.**
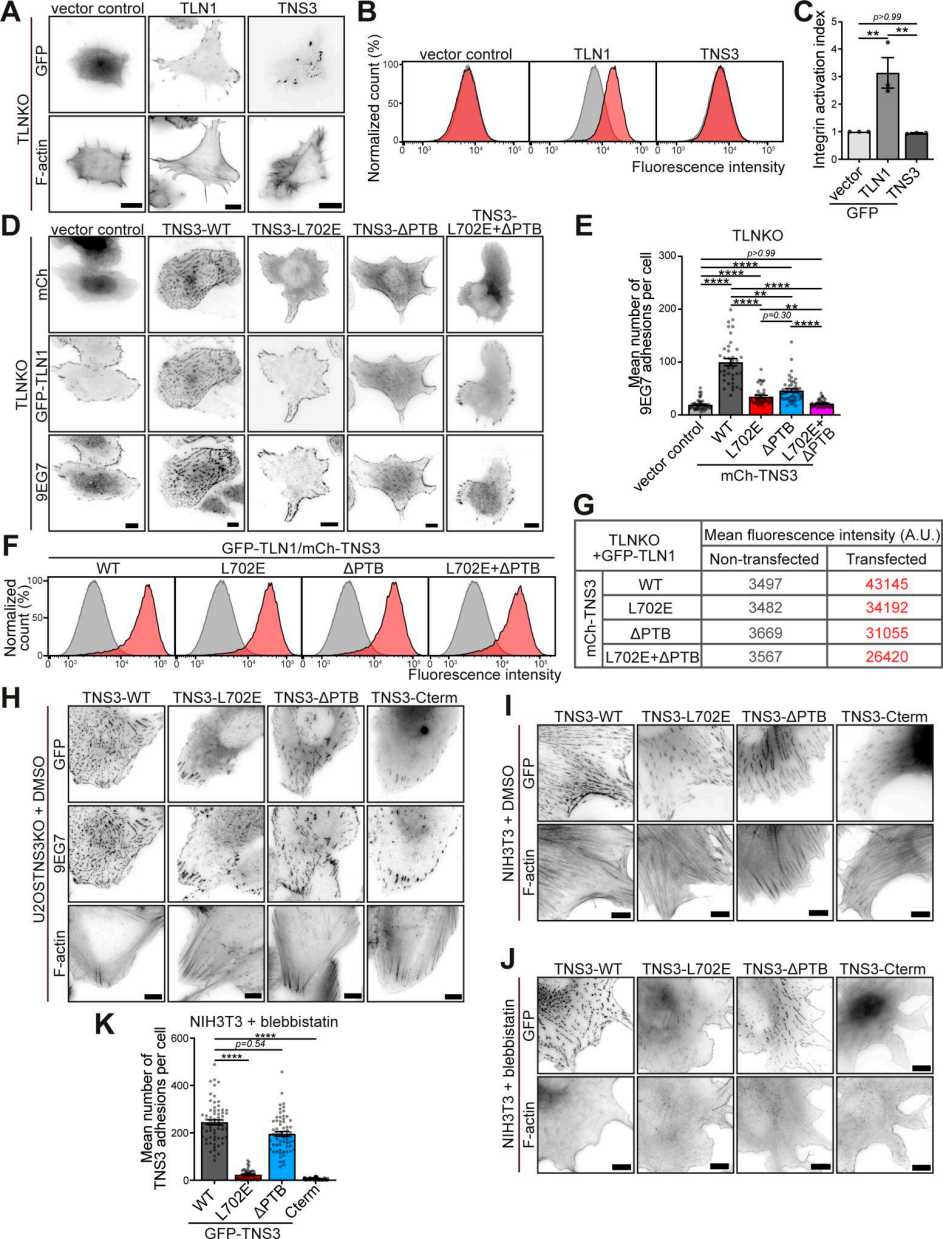
**Tensin3 regulates β1 integrin activity in the presence of talin. (A)** Images of TLNKO cells expressing GFP-vector control, GFP-TLN1, or GFP-TNS3. F-actin was visualized by phalloidin staining. **(B)** Representative integrin activation (β1) profiles of TLNKO cells expressing GFP-vector, GFP-TLN1, or GFP-TNS3 as measured by flow cytometry analysis. Red profiles are from cells expressing the indicated constructs, and gray profiles are from the non-transfected cells in the same samples. **(C)** Integrin activation index (normalized to cells expressing GFP-vector) calculated from triplicate experiments of B. **(D)** Representative images of TLNKO cells co-expressing GFP-TLN1 with mCh-vector or mCh-TNS3 constructs (shown in Fig. 5 E), respectively. Activated β1 integrin was visualized by staining with 9EG7 antibody. **(E)** Quantification of 9EG7-positive adhesions in D pooled from three independent experiments; *n* = 42 (vector), 38 (WT), 41 (L702E), 46 (ΔPTB), and 40 (L702E+ΔPTB) cells. **(F)** Representative integrin activation profiles of TLNKO cells co-expressing GFP-TLN1 with different mCh-TNS3 constructs. **(G)** Mean fluorescence intensity of F. Red values are from transfected cells, and gray values are from the nontransfected cells in the same samples. Note that the quantification of the integrin activation index pooled from three replicates is shown in Fig. 5 H. **(H)** Representative images of U2OS TNS3KO cells expressing GFP-TNS3 constructs (WT, L702E, ΔPTB, or L702E+ΔPTB). Cells were cultured on FN-coated glass overnight before being treated with DMSO or blebbistatin (50 μM, shown in Fig. 5 J) for 60 min. Actin and β1 integrin were visualized by staining with phalloidin and 9EG7 antibody. Note that all four GFP-TNS3 constructs were localized to adhesions when cells were treated with DMSO. **(I and J)** NIH3T3 cells transfected with GFP-TNS3 constructs (same as those used in H) were treated with DMSO (I) or blebbistatin (50 μM, J) for 60 min before fixation and stained for actin. **(K)** Quantification of GFP-TNS3–positive adhesions in J. Note that GFP-TNS3-WT– and GFP-TNS3-ΔPTB–positive adhesions largely remain after blebbistatin treatment, whereas GFP-TNS3-L702E–positive and GFP-TNS3-Cterm–positive adhesions mostly disappear. *n* = 64 (WT), 49 (L702E), 62 (ΔPTB), and 71 (Cterm) cells. All error bars are the SEM. ** indicates P < 0.01, and **** indicates P < 0.0001 (C: ordinary one-way ANOVA with Turkey’s multiple comparisons, E and K: Kruskal–Wallis test with Dunn’s multiple comparisons test). Data are collected from three independent experiments. Scale bars in A, D, and H–J, 10 μm.

**Figure 5.**
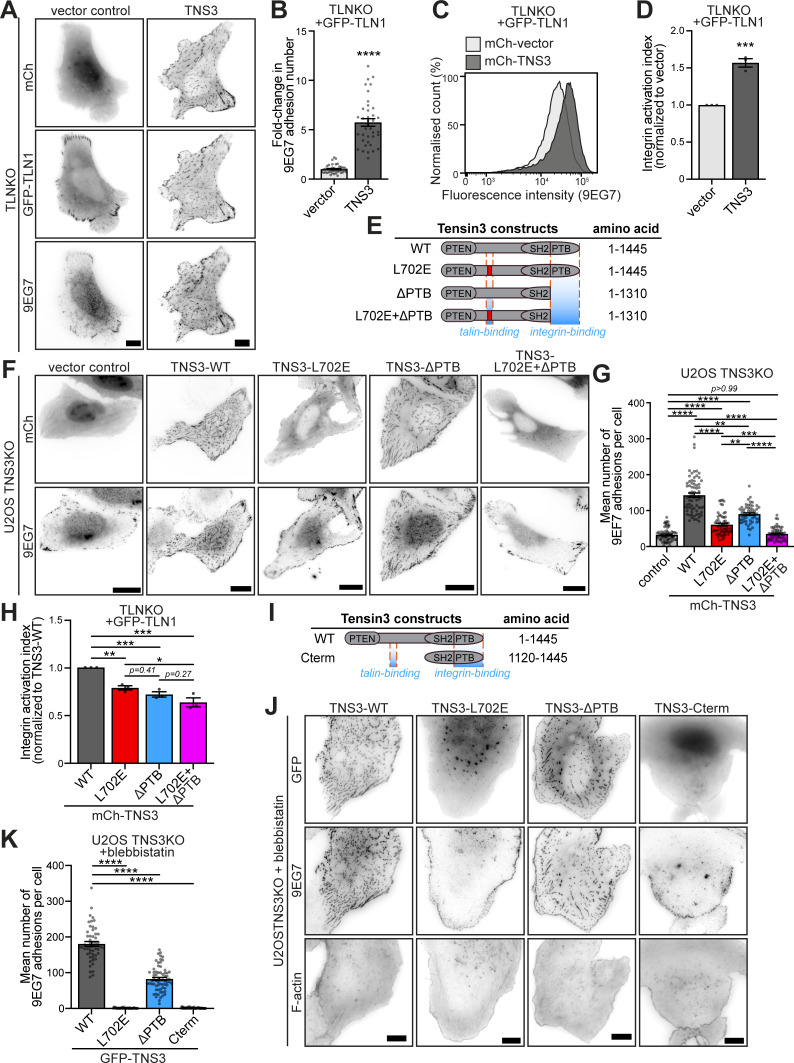
**Tensin3 regulates integrin activity through its interaction with talin and integrin. (A)** Representative images of TLNKO cells co-expressing GFP-TLN1 with mCh-vector or mCh-TNS3, respectively. Active β1 integrins were stained with 9EG7 antibody. **(B)** Quantification of the fold change in 9EG7-positive adhesion counts in A. The fold change was calculated by normalizing the values for cells expressing mCh-TNS3 to the mean value for cells expressing mCh-vector. **(C)** Representative integrin activation (β1, 9EG7 antibody) profiles of TLNKO cells co-expressing GFP-TLN1 with mCh-vector (light gray) or mCh-TNS3 (dark gray) as measured by flow cytometry. **(D)** Integrin (β1) activation index (normalized to cells expressing GFP-TLN1 and mCh-vector) calculated from triplicate experiments of C. **(E)** Schematic of the tensin3 deletion and mutation constructs used, with the TBS motif (red) and the integrin-binding PTB domain indicated by orange dashed lines. All constructs are N-terminally labeled with GFP or mCherry. **(F)** Images of U2OS TNS3KO cells expressing the mCh-TNS3 construct shown in E, or a vector control. Cells were plated overnight on FN-coated glass-bottom dishes and stained for active β1 integrin (9EG7). **(G)** Quantification of β1 integrin–positive adhesions in F. *n* = 66 (vector), 70 (WT), 58 (L702E), 55 (ΔPTB), and 58 (L702E+ΔPTB) cells. **(H)** Integrin activation index (normalized to cells expressing GFP-TLN1 and mCh-TNS3-WT) calculated from triplicate flow cytometry experiments of Fig. S3, F and G. **(I)** Schematic of the tensin3 C-terminal deletion construct used. Note that this construct is N-terminally labeled with GFP. **(J)** U2OS TNS3KO cells expressing GFP-TNS3 constructs shown in E and I were cultured overnight before treatment with blebbistatin (50 μM) or an equivalent volume of DMSO (shown in Fig. S3 H) for 60 min. **(K)** Quantification of β1 integrin–positive adhesion in J. *n* = 53 (WT), 43 (L702E), 66 (ΔPTB), and 53 (Cterm) cells. All data are collected from three independent experiments. Scale bars: 10 μm (A and J); 20 μm (F). Error bars are the SEM; * indicates P < 0.05, ** indicates P < 0.01, *** indicates P < 0.001, **** indicates P < 0.0001 (B, D: unpaired *t* test; G and K: Kruskal–Wallis test with Dunn’s multiple comparisons test; H: ordinary one-way ANOVA with Turkey’s multiple comparisons).

Cells without talin do not spread, but re-expression of talin1, due to its ability to activate integrins (Zhang et al., 2008), rescues both cell adhesion formation and cell spreading (Atherton et al., 2015). Tensins also interact with integrins via their PTB domain and are thought to contribute to integrin activation (Calderwood et al., 2003; Torgler et al., 2004). The experiments shown in Fig. 4 revealed that the interaction of tensin3 with talin was the driving force for the phenotypic changes in the centrally located adhesion sites. To gain a deeper insight into the mechanisms, we tested their contribution to integrin activation in more detail. Since the exogenous expression of tensin3 alone in TLNKO cells did not rescue cell spreading (Fig. S3 A) or integrin activation (Fig. S3, B and C), it is evident that tensin3 cannot substitute for talin. In a second set of experiments, we quantified adhesion formation in TLNKO cells co-expressing GFP-TLN1 together with mCh-TNS3 and compared this with adhesion in cells rescued by GFP-TLN1 and vector control (mCh-vector, Fig. 5 A). Interestingly, staining of active integrins with a conformation-sensitive antibody (Lenter et al., 1993) showed that cells co-expressing talin1 and tensin3 exhibited a fivefold increase in adhesion sites compared with those expressing talin1 and vector control (Fig. 5 B). A similar result of tensin3-induced increased integrin activation was observed using flow cytometry (Fig. 5, C and D). The question arose as to whether such additive effects of integrin activation were due to tensin3 binding to integrins or to a scenario in which tensin3 binding to talin stabilized talin in an active conformation.

To address these questions, we generated a series of tensin3 constructs (Fig. 5 E), including one lacking the reported integrin-binding PTB domain (mCh-TNS3-ΔPTB) and the same construct carrying the L702E point mutation that blocks the interaction with talin (mCh-TNS3-L702E+ΔPTB). While the expression of TNS3-WT in U2OS TNS3KO fully rescued adhesion formation (Fig. 5, F and G), the adhesion-promoting effect of tensin3 was significantly reduced when cells expressed either TNS3-ΔPTB (37%) or TNS3-L702E (57%). Cells expressing a tensin construct that lacks both the ability to bind integrins and talin (mCh-TNS3-L702E+ΔPTB) could not rescue adhesion formation and showed a similar phenotype to cells expressing the control vector (Fig. 5, F and G). Similar results were observed in TLNKO cells (Fig. S3, D and E) where GFP-TLN1 was co-expressed with different mCh-TNS3 constructs. Furthermore, flow cytometry showed that integrin activity in TLNKO cells expressing WT forms of talin and tensin3 (Fig. 5 H; and Fig. S3, F and G) was significantly reduced by loss of the talin–tensin3 interaction (L702E; 21%) or the tensin3 integrin-binding PTB domain (ΔPTB; 28%). It was further reduced by eliminating both the talin–tensin3 interaction and the tensin3 PTB domain (L702E+ΔPTB; 36%).

Taken together, these results demonstrate that tensin3 contributes to integrin activity through its PTB domain and through its interaction with talin.

### Tensin3 binding to integrin is not essential for the formation of stable FBs

One of the hallmarks of FBs is that once they have matured from FAs, their maintenance becomes independent of actomyosin-mediated tension (Atherton et al., 2022). To understand how tensin3 might contribute to such adhesion stability, we expressed our various tensin constructs (Fig. 5 I) in U2OS TNS3KO cells, plated them on FN to allow the formation of tensin3 adhesions, and then treated them with the actomyosin inhibitor blebbistatin (50 µM, Fig.5 J). Cells expressing GFP-TNS3-WT exhibited a large number of β1 integrin-positive adhesions (Fig. S3 H), which remained present despite actomyosin inhibition (Fig. 5 J). Interestingly, the expression of GFP-TNS3-ΔPTB, but not the talin-binding mutant GFP-TNS3-L702E, was able to stabilize adhesion sites under these conditions (Fig. 5, J and K). The expression of the integrin-binding C terminus comprising the SH2 and PTB regions of tensin (GFP-TNS3-Cterm, Fig. 5 J) was also unable to stabilize adhesions when expressed in TNS3KO cells. Similar observations were made when tensin constructs were expressed in NIH3T3 cells (Fig. S3, I–K), confirming that the tensin3–talin interaction is critical for the formation of stable, force-independent FBs. However, the presence of the integrin-binding PTB domain appeared to be largely irrelevant in this process.

Taken together, these results demonstrate that although tensin3 alone cannot induce integrin activation, it is essential for forming stable FBs in the absence of actomyosin-mediated tension. Interestingly, the PTB domain of tensin3, which binds directly to integrins (Calderwood et al., 2003; McCleverty et al., 2007), is not necessary for the sustained integrin activity in FBs.

### Tensin3 undergoes LLPS in cells

**Figure S4.**
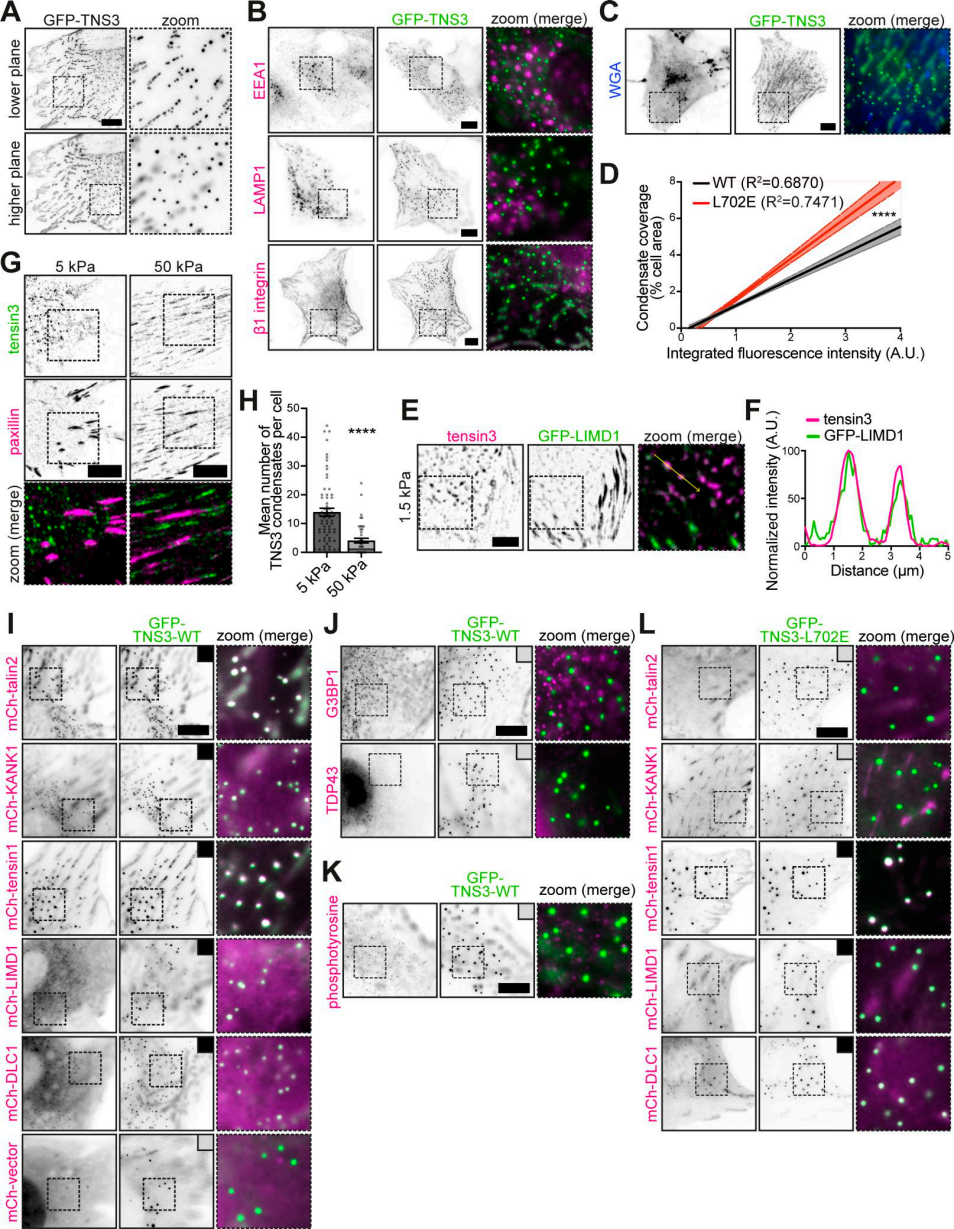
**Talin is a retention signal for tensin3 that controls mechanosensitive tensin3 condensation and the client protein recruitment. (A)** Representative images of NIH3T3 cells expressing GFP-TNS3 at a lower (0 μm) and a higher (0.6 μm) focal plane, with dashed boxes zoomed to the right. Note that TNS3 spheres are observed in proximity to the adhesion plane and in the cytoplasm. **(B)** Representative images of NIH3T3 cells expressing GFP-TNS3 (green) with immunostaining of endosomal marker EEA1, lysosomal marker LAMP1, or active β1 integrins (magenta). **(C)** Images of an NIH3T3 cell forming GFP-TNS3 condensates, labeled with fluorescently conjugated WGA (shown in blue). **(D)** Correlation between the relative protein level of mCh-TNS3-WT or mCh-TNS3-L702E and the cellular coverage (%) of TNS3 condensates in NIH3T3 cells, represented by linear regressions with 95% confidence intervals. *n* = 181 cells (WT) and 168 cells (L702E); nonparametric Spearman’s correlation r = 0.8773 (WT) and 0.9013 (L702E). **(E)** Representative background-subtracted images of a HFF cell expressing GFP-LIMD1 with staining for endogenous tensin3. The dashed box is zoomed in on the right, with a yellow arrow above two tensin3 condensates. **(F)** Line profile for the yellow arrow in E. **(G)** Background-subtracted images of HFF cells plated overnight on FN-coated 5 or 50 kPa PAA hydrogels. Endogenous tensin3, green; paxillin, magenta. **(H)** Quantification of the mean condensate number in G. *n* = 40 (5 kPa) and 41 (50 kPa) cells. **(I)** Representative images of NIH3T3 cells expressing GFP-TNS3-WT in green with exogenously co-expressed proteins in magenta. The black box indicates recruitment to the TNS3 condensates, and the gray box indicates no recruitment. **(J and K)** Images of NIH3T3 cells forming the TNS3 condensates in green with immunofluorescence staining for stress granule protein G3BP1 and TDP43 (J), and for tyrosine-phosphorylated proteins (K) with an antibody that probes phosphotyrosine (clone 4G10) in magenta. **(L)** Images of NIH3T3 cells forming the TNS3-L702E condensates in green with exogenously co-expressed proteins in magenta. Note that the summary table for I–L is shown in Fig. 8 C. **** indicates P < 0.0001 (D: ANCOVA; H: Mann–Whitney test). Scale bars are 10 µm (A–C, G, and I–L) or 5 µm (E). ANCOVA, analysis of covariance.

**Figure 6.**
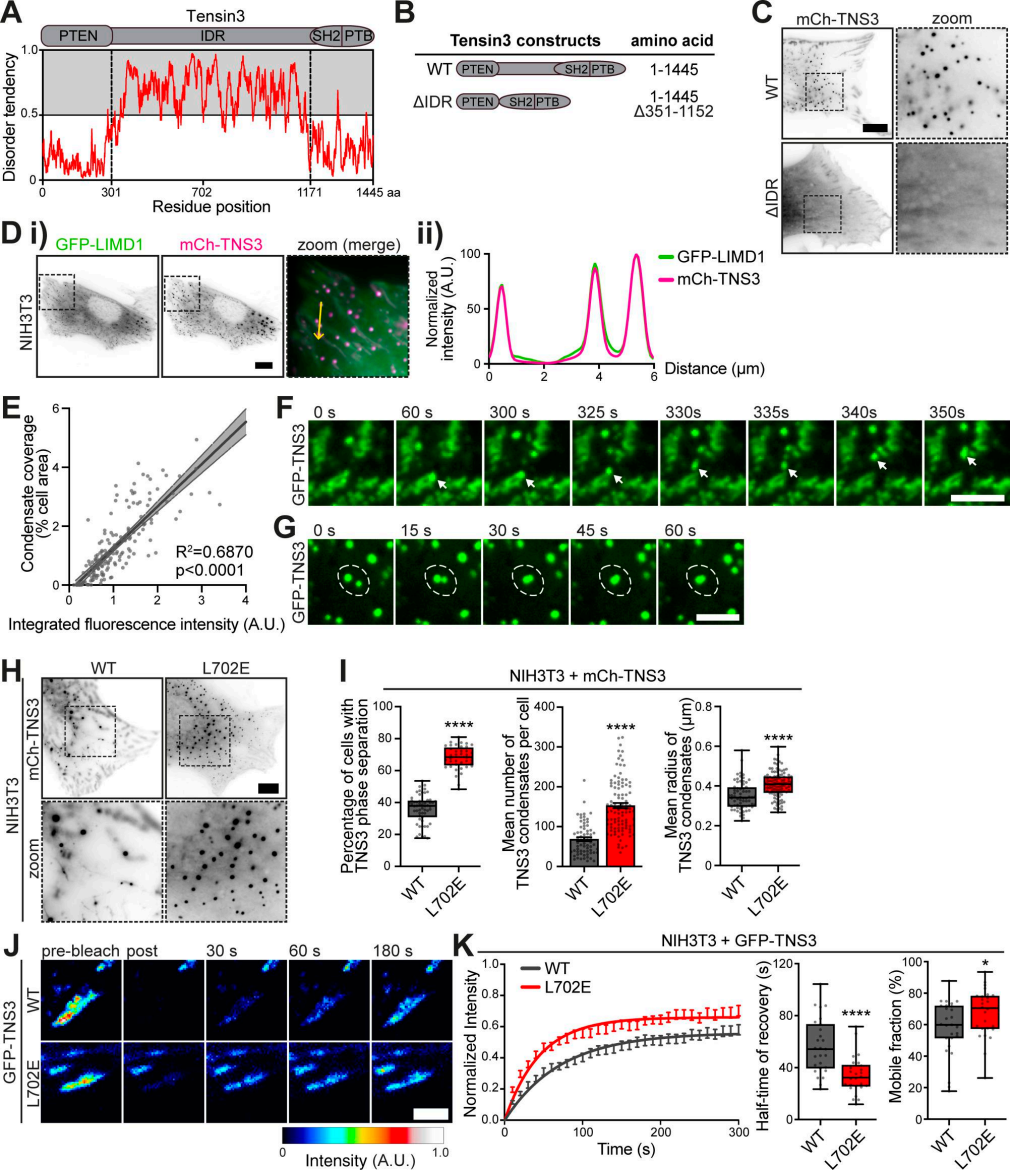
**Tensin3 undergoes LLPS in cells in a talin-regulated manner. (A)** Schematic of human tensin3 with predicted disorder degree by IUPred (Dosztányi, 2018). Residues with a predicted disorder tendency higher than 0.5 are considered disordered. **(B)** Schematic of the tensin3 IDR deletion (ΔIDR, deletion of aa 351–1,152) construct used. Note that this construct is N-terminally labeled with mCh. **(C)** Representative images of NIH3T3 cells expressing mCh-TNS3-WT or mCh-TNS3-ΔIDR. The dashed boxes are zoomed in on the right. Note that deletion of the IDR abolished the formation of tensin3 condensates. **(D i and ii)** Image (i) of NIH3T3 cells co-expressing GFP-LIMD1 (green) and mCh-TNS3 (magenta). The dashed box is zoomed in on the right (ii), with a line profile below for the yellow arrow line. **(E)** Correlation between the relative protein level of mCh-TNS3 and the cellular coverage (%) of TNS3 condensates in individual NIH3T3 cells, represented by a linear regression with 95% confidence intervals. *n* = 181 cells; R^2^ = 0.6870; r = 0.8773 (nonparametric Spearman’s correlation). **(F)** Time-lapse images of a small GFP-TNS3 condensate (indicated by white arrows) derived from adhesion sites in NIH3T3 cells, related to Video 1. **(G)** Time-lapse images of GFP-TNS3 condensates (highlighted by dashed white circles) fusing in NIH3T3 cells, related to Video 2. **(H)** Images of NIH3T3 cells expressing mCh-TNS3-WT or mCh-TNS3-L702E. **(I)** Quantification of H pooled from triplicate experiments. Note that condensates that are larger than 0.1 μm^2^ with a circularity between 0.7 and 1 were quantified. Left panel: The percentage of transfected cells with TNS3 condensate formation in each area (610 × 499 μm). The boxes represent the 25–75th percentiles with the median indicated; the whiskers indicate the range of values; *n* = 56 (WT) and 41 (L702E) areas. Middle panel: The mean number of TNS3 condensates in cells. Error bars are the SEM. Right panel: The mean radius of TNS3 condensates in each cell. *n* = 68 (WT) and 100 (L702E) cells. **(J)** Time-lapse images of FRAP experiment in NIH3T3 cells expressing GFP-TNS3-WT or GFP-TNS3-L702E. **(K)** Quantification of recovery halftime (left and middle panels) and mobile fraction (right panel) in J pooled from triplicate experiments. *n* = 27 (WT, 15 cells) and 26 (L702E, 17 cells) adhesions. * indicates P < 0.05, and **** indicates P < 0.0001 (I right and left panel: Unpaired *t* test; I middle panel and K: Welch’s *t* test). Scale bar: 10 µm (C, D, and H); 5 μm (F and G); 3 μm (J).

During our investigations, we frequently observed tensin3-positive spherical structures, particularly in the cytoplasm (Fig. S4 A), upon exogenous expression of tensin3. These structures were negative for endosomal marker EEA1, lysosomal marker LAMP1, and active β1 integrins (Fig. S4 B), excluding the possibility that these structures are involved in matrix engulfing endocytic vesicles (as previously observed for tensin1 [Rainero et al., 2015]), or degradation processes. Recent reports have emphasized the possibility of proteins that contain IDRs undergoing LLPS (Banani et al., 2017; Wright and Dyson, 2015), a process of protein condensation driven by multivalent interactions between molecules (Li et al., 2012). Since tensin3 has an extended IDR (Fig. 6 A), we speculated that the observed tensin3 spheres were LLPS condensates. Eliminating the IDR (mCh-TNS3-ΔIDR) completely abolished the formation of tensin3 spheres (Fig. 6, B and C). A hallmark of such LLPS condensates is that they are membraneless. To test this, we stained the observed structures using the membrane marker wheat germ agglutinin (WGA) (Chang et al., 1975), which confirmed that they were negative for lipid membranes (Fig. S4 C). Further data showed that the mCh-TNS3 spheres colocalized perfectly with GFP-LIMD1 (Fig. 6 D), another protein reported to undergo LLPS (Wang et al., 2021). Since LLPS is highly concentration-dependent, we analyzed the condensate formation in cells expressing various levels of mCh-TNS3 and observed a strong positive correlation (Fig. 6 E). Moreover, time-lapse recordings of GFP-TNS3 expressed in NIH3T3 cells revealed the release of small spheres from adhesion sites into the cytoplasm (Fig. 6 F and Video 1), which, as expected for liquid-like structures, fused into larger circular spheres (Fig. 6 G and Video 2).

**Video 1.** **Small GFP-TNS3 condensate derived from adhesion sites, related to** Fig. 6 F **.** Time-lapse movie of GFP-TNS3 condensate formation at adhesion sites and release into the cytoplasm in a NIH3T3 cell. The green box indicates the zoomed area in Fig. 6 F. Time interval: 5 s; total time: 5 min 50 s. Scale bar: 10 µm.

**Video 2.** **Dynamic fusion event of GFP-TNS3 condensates, related to** Fig. 6 G **.** Movie of exogenously expressed tensin3 condensates fusing into larger spheres in a NIH3T3 cell. The green box indicates the zoomed area in Fig. 6 G. Time interval: 5 s; total time: 5 min 15 s. Scale bar: 10 µm.

Taken together, these data show that tensin3 undergoes LLPS when exogenously expressed in cells.

### Talin regulates the formation of tensin3 condensates

During the course of our experiments, we observed that the tensin3 bearing the talin-binding mutation (L702E) formed higher numbers of LLPS condensates in cells. Quantification revealed a dramatic 2.2-fold increase in the number of condensates formed by TNS3-L702E compared with those formed by TNS3-WT (Fig. 6, H and I). Condensates formed by TNS3-L702E were also larger than those found in cells expressing TNS3-WT (Fig. 6 I, right panel), suggesting more frequent fusion events of condensates from the talin-binding mutant. Correlation analysis of condensate coverage and cellular TNS3 expression revealed that L702E significantly increases the propensity for TNS3 condensation in cells (Fig. S4 D). Most strikingly, TNS3-WT expressed in U2OS TNS3 KO cells remained in adhesion structures when treated with blebbistatin, whereas TNS3-L702E was almost exclusively found in the LLPS condensates in the cytoplasm (see Fig. 5 J, top row, panels 1 and 2). These data suggest that talin retains tensin3 in cell–matrix adhesions by binding to tensin3 and that disruption of this interaction promotes tensin3 LLPS.

If talin acts as a retention signal for tensin3 in adhesions, one would expect that the lack of talin binding would result in a decreased residence time (mobility) of tensin3 in adhesion sites. To assess this, we measured tensin3 mobility by performing fluorescence recovery after photobleaching (FRAP) experiments (Fig. 6 J). As expected, both the halftime of recovery and the mobile fraction of TNS3-L702E were significantly higher than those of TNS3-WT (Fig. 6 K), demonstrating increased mobility of tensin3 that cannot bind talin in adhesions.

Taken together, these data show that talin controls the dynamics of tensin3 and its propensity to undergo LLPS through direct interaction.

### Tensin3 LLPS is controlled by talin activity in response to rigidity sensing

**Figure 7.**
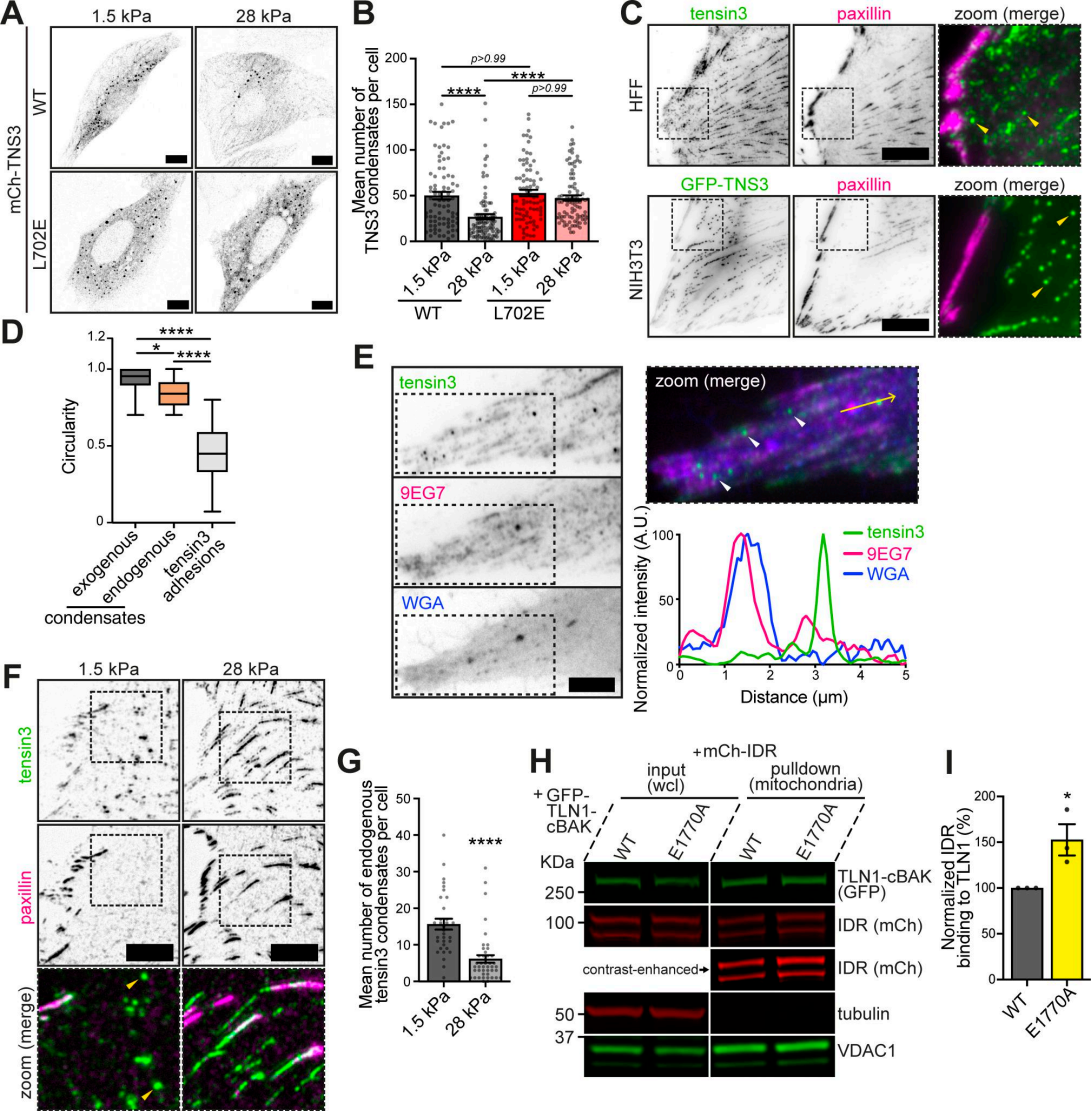
**Tensin3 LLPS is controlled by talin activity in response to rigidity sensing. (A)** Confocal images of NIH3T3 cells expressing mCh-TNS3-WT or mCh-TNS3-L702E plated overnight on FN-coated 1.5 or 28 kPa PDMS dishes. **(B)** Quantification of the mean TNS3 condensate number in A. *n* = 83 (WT/1.5 kPa), 80 (WT/28 kPa), 83 (L702E/1.5 kPa), and 89 (L702E/28 kPa) cells. Note that condensates that are larger than 0.1 μm^2^ with a circularity between 0.7 and 1 were quantified. **(C)** Images of endogenous tensin3 or GFP-TNS3 (green) with endogenous paxillin (magenta) in HFF cells (top panel) and NIH3T3 cells (bottom panel) plated on FN-coated glass, respectively. The dashed boxes are zoomed to the right, with condensates indicated by yellow arrows. **(D)** Quantification of the circularity of overexpressed GFP-TNS3 condensates in NIH3T3 cells, endogenous tensin3 condensates in HFF cells, and tensin3 adhesions in HFF cells. The boxes represent the 25–75th percentiles with the median indicated; the whiskers indicate the range of values. *n* = 717 (overexpressed condensates, 17 cells), 192 (endogenous condensates, 29 cells), and 4,883 (tensin3 adhesions, 33 cells). **(E)** Representative images of HFF cells plated on FN-coated glass (left panel). The dashed boxes are zoomed to the right panel, with tensin3 condensates (green) indicated by white arrows and a line profile below for the yellow arrow line. The lipid membrane is labeled with WGA (blue); the active β1 integrin (magenta) is stained using 9EG7 antibody. Note that the tensin3 condensate is not colocalized with vesicle structures indicated by WGA and active β1 integrin. **(F)** Images (background-subtracted) of HFF cells plated overnight on FN-coated 1.5 or 28 kPa PDMS dishes. Endogenous tensin3 (green) and paxillin (magenta) were visualized by staining. The dashed boxes are zoomed in below, with condensates indicated by yellow arrows. **(G)** Quantification of the mean number of endogenous tensin3 condensates in F. *n* = 33 (1.5 kPa) and 40 (28 kPa) cells. **(H)** Mitochondrial pulldown experiments using HEK293T cells expressing GFP-TLN1-cBAK (WT or E1770A) and mCh-IDR. **(I)** Quantification of H pooled from three replicates. Data are normalized to WT. Error bars are the SEM. All data are collected from three independent experiments. * indicates P < 0.05, and **** indicates P <0.0001 (B and D: Kruskal–Wallis test with Dunn’s multiple comparisons; G: Mann–Whitney test; I: unpaired *t* test). Scale bar: 10 µm (A, C, and F) and 5 µm (E). Source data are available for this figure: SourceData F7.

While talin is critically involved in mechanosensing (Goult et al., 2018; Goult et al., 2021), forces have been shown to contribute to its activation state (del Rio et al., 2009; Yao et al., 2016). The residence time of talin in FAs increases when cells encounter stiff substrates (Stutchbury et al., 2017), as it has been shown for constitutively active talin constructs (Atherton et al., 2020). This led to the hypothesis that talin activity can regulate tensin3 retention in a tension-dependent manner, which subsequently modulates tensin3 LLPS. To test this hypothesis, we expressed mCh-TNS3 in NIH3T3 cells and plated them on FN-coated elastic polydimethylsiloxane (PDMS) surfaces of different stiffness (Fig. 7 A). The results show a significant twofold increase in TNS3-WT condensates in cells plated on soft (1.5 kPa) substrates compared with those plated on stiff (28 kPa) substrates (Fig. 7 B). Interestingly, the TNS3-L702E, which cannot bind talin, showed no difference in condensate formation on soft versus stiff substrates. This supports a model in which the change in tensin3 phase separation in respond to the substrate environment is mediated by talin.

While the above observations were made in cells exogenously expressing varying levels of tensin3, we wondered whether endogenous tensin3 could form similar condensates. We therefore performed immunostaining for tensin3 in primary human foreskin fibroblasts (HFFs), which contain substantial levels of tensin3. Unlike paxillin that localized exclusively to FAs in these cells, tensin3 also localized in circular structures as observed in cells expressing low levels of GFP-TNS3 (Fig. 7, C and D). Similar to those condensates, endogenous structures were negative for integrins and for the plasma membrane marker WGA (Fig. 7 E). Comparing the endogenous structures on substrates of different rigidity, we found a significantly increased number (3.6-fold) of tensin3 spheres in cells plated on soft substrates (1.5 kPa, Fig. 7, F and G) versus those plated on stiff substrates (28 kPa). Similar observations were made with soft (5 kPa) and stiff (50 kPa) polyacrylamide (PAA) hydrogels (Fig. S4, G and H). Such small tensin condensates (Fig. 7 F) weakly colocalize with GFP-LIMD1 upon the expression of the latter (Fig. S4, E and F). Together, these results demonstrate that endogenous tensin3 forms similar condensates to exogenously expressed tensin3, which share the same mechanosensitive pattern.

Since talin can be mechanically activated, we thought the increased retention of tensin3 could be due to talin changing its activation status when encountering substrates of different stiffness (Elosegui-Artola et al., 2016). Such mechanism is known for the talin–vinculin interaction, whereby increased substrate stiffness could lead to increased tension on talin with subsequent activation of previously cryptic vinculin-binding sites (Yao et al., 2016). To test whether the activation state of talin alters tensin3 binding, we performed mitochondrial pulldown experiments using a constitutively active TLN1-E1770A-cBAK construct co-expressed with TNS3-IDR and compared it with that of TLN1-WT-cBAK (Fig. 7 H). Interestingly, constitutively active talin (E1770A) increases IDR pulldown by 50% compared with WT talin (Fig. 7 I), demonstrating that activated talin has an increased binding capacity to tensin3.

### Tensin3 LLPS compartmentalizes adhesion proteins and provides a platform for signaling components

**Figure 8.**
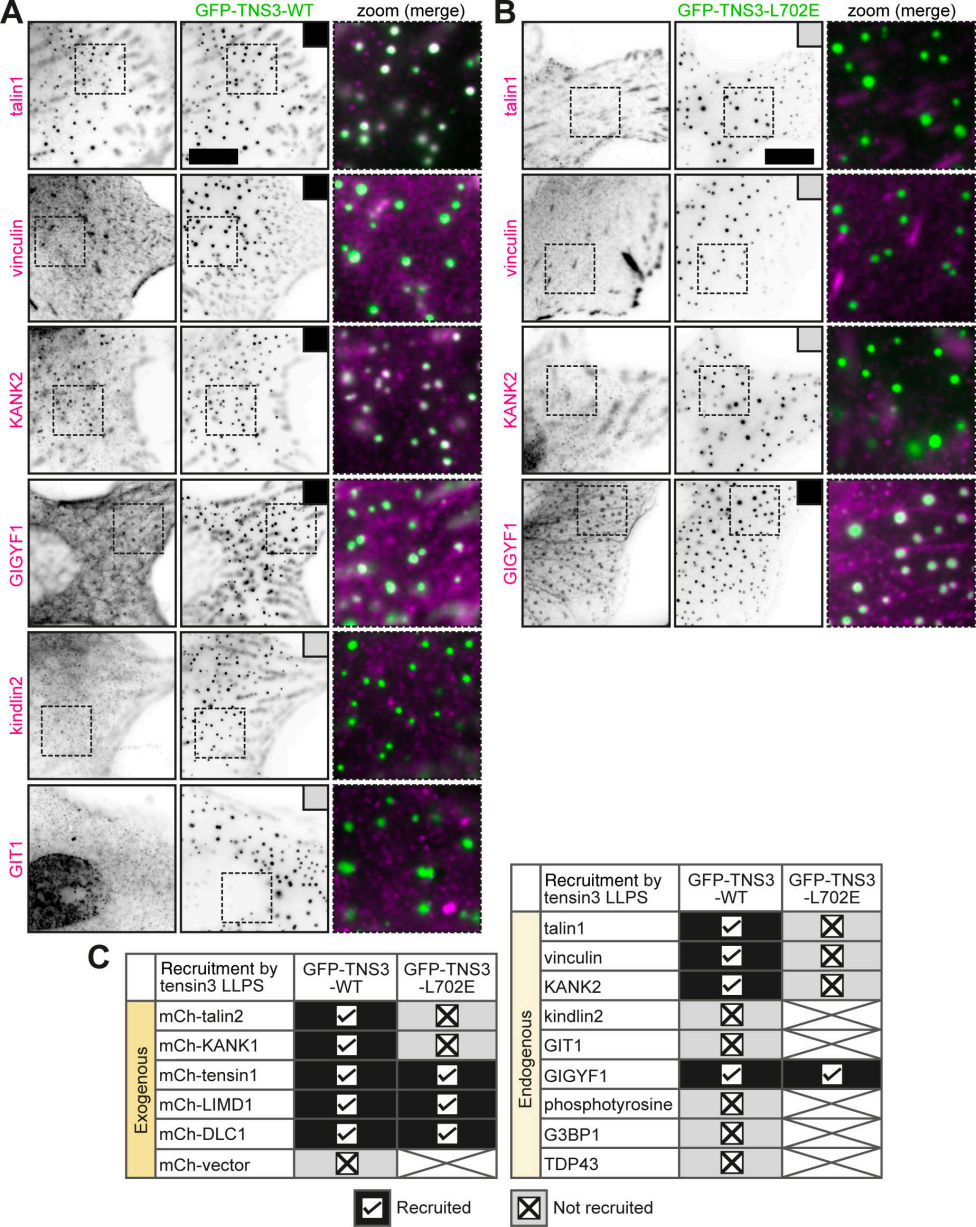
**Tensin3 LLPS compartmentalizes adhesion and signaling proteins. (A and B)** Representative images of NIH3T3 cells forming GFP-TNS3-WT (A) or GFP-TNS3-L702E (B) condensates (green) with immunostaining of various proteins (magenta). The dashed boxes are zoomed to the right. The black box indicates the recruitment to condensates, and the gray box indicates no recruitment. Scale bars are 10 µm. **(C)** Summary table of colocalization analysis of exogenously expressed (left panel, images are included in Fig. S4, I and L) or endogenous proteins (right panel, images are shown in Fig. 8, A and B; and Fig. S4, J and K) with GFP-TNS3-WT or GFP-TNS3-L702E condensates in NIH3T3 cells.

Since the observed tensin3 condensates were derived from adhesion sites in a talin-regulated mechanosensitive manner, we wondered whether these condensates contain other adhesion components or signaling molecules. To address this question, we examined a selection of cell–matrix adhesion proteins that have been reported by others to be present in LLPS condensates. These proteins include LIMD1 (Wang et al., 2021), kindlin2 (Hsu et al., 2023), talin (Hsu et al., 2023; Litschel et al., 2024), KANK (Guo et al., 2023), tensin1 (Lee et al., 2023), vinculin (Litschel et al., 2024), and GIT1 (Zhu et al., 2020). We added the actin regulatory RhoGAP DLC1 to this selection, as it is known to bind tensin (Liao et al., 2006; Shih et al., 2015), and the RNA regulatory protein GIGYF1, which we had previously identified alongside many of the aforementioned proteins in tensin BioID datasets (Atherton et al., 2022). We also added stress granule components G3BP1 (Tourrière et al., 2023) and TDP-43 (Colombrita et al., 2009). These proteins were either co-expressed as fluorescently tagged proteins (Fig. S4 I) or, where antibodies were available, probed endogenously in cells that formed GFP-TNS3 condensates (Fig. 8 A). Proteins that were detected in tensin3 condensates were the actin cross-linking proteins talin1 and talin2, vinculin, tensin1, and LIMD1, together with the microtubule-targeting protein KANK1 and KANK2, and DLC1 and GIGYF1 (Fig. 8 A and Fig. S4 I). In contrast, kindlin2 and GIT1, which regulate membrane protrusion (Böttcher et al., 2017; Manabe et al., 2002), were absent from the tensin3 condensates (Fig. 8 A). These condensates were also negative for G3BP1, TDP-43 (Fig. S4 J), or tyrosine phosphorylation (Fig. S4 K).

Next, we asked whether the tensin3–talin interaction controls the talin recruitment into the tensin3 condensates. Therefore, we repeated the colocalization experiments in cells expressing the talin-binding mutant TNS3-L702E (Fig. 8 B and Fig. S4 L). Under these conditions, not only talin1/2, but also the talin-binding partner KANK1/2 (Bouchet et al., 2016; Sun et al., 2016) and vinculin (Gingras et al., 2005), failed to localize into the tensin3 condensates. In contrast, tensin1, LIMD1, DLC1, and GIGYF1 remained present (Fig. 8 C).

These data demonstrate that tensin3 LLPS can drive the compartmentalization of adhesion proteins and signaling molecules. During the LLPS of tensin3, the TBS remains accessible, which directly recruits talin and indirectly recruits KANK and vinculin into the tensin3 condensates.

## Discussion

Here, we have elucidated how talin interacts with tensin3 to coordinate FB formation and FN remodeling. We identified four tensin3-binding sites in the talin rod domains R3, R4, R8, and R11 (Fig. 1), all of which engage the same TBS in tensin3. Determining the structure of the tensin3 TBS in complex with talin R11R12 (Fig. 2, A–C) enabled us to design a point mutation (L702E) in tensin3 that completely disrupted its interaction with talin. Structural insight also allowed us to design mutations that selectively disrupted talin R8 and R11 binding to tensin3. These specific residues were identified as being critical for cells to form tensin3-dependent FBs and for FN fibrillogenesis (Fig. 4, A–D). Intriguingly, we found that talin interaction also regulates the LLPS of tensin3 (Fig. 6, H and I; and Fig. S4 D). Perturbing the talin–tensin3 interaction not only altered the mechanosensitive formation of tensin3 condensates, but also changed their composition (Fig. 8 C).

**Figure S5.**
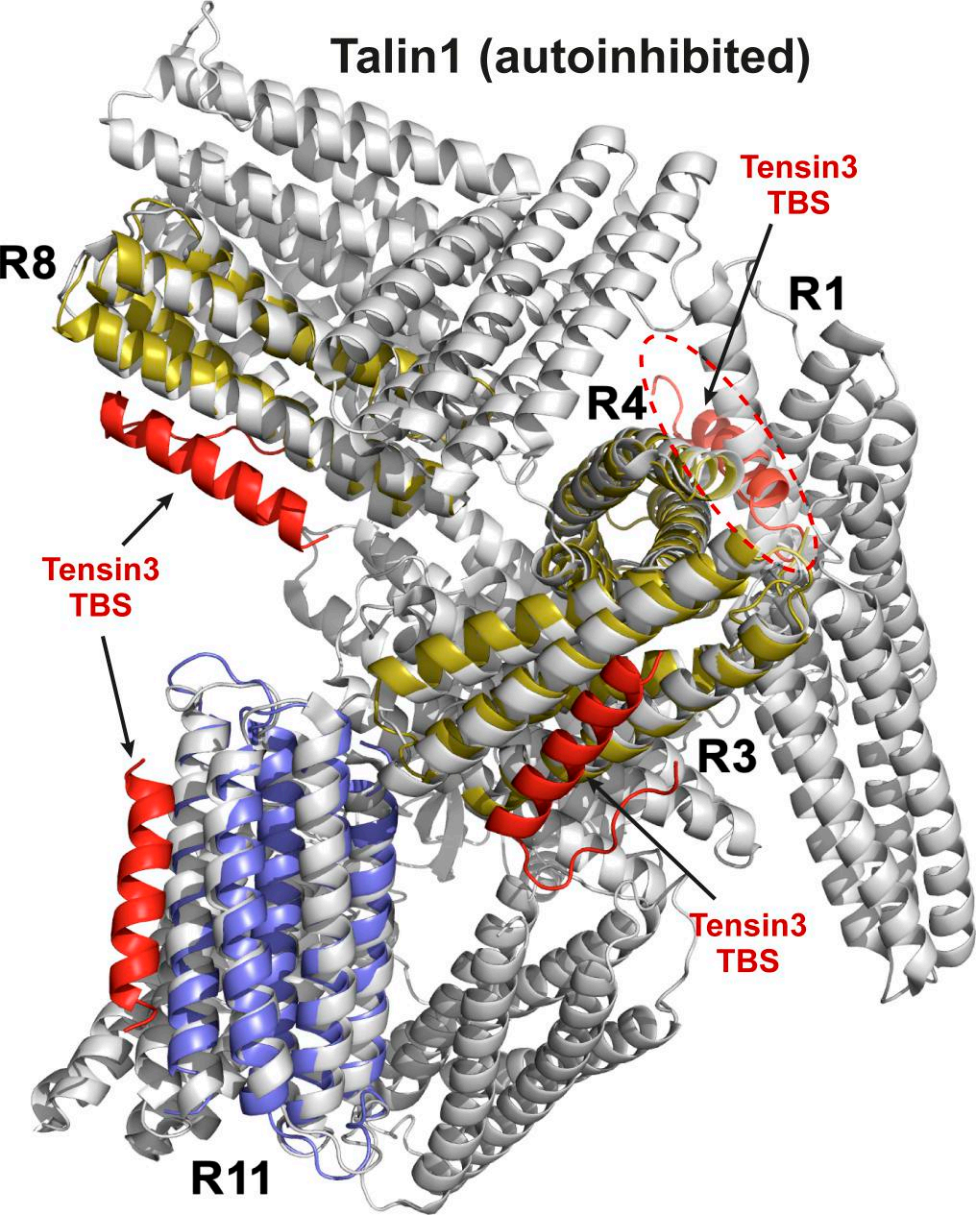
**Schematic of tensin3 TBS binding to the autoinhibited talin1.** Superposition of the NMR-validated AlphaFold3 models of the talin R3, R4, and R8 (yellow), and crystal structure of the talin R11 (purple) in complex with tensin3 TBS peptide (red) on the cryoEM structure of the autoinhibited form of the full-length talin1 (PDB ID: 8VDQ, gray). The superposition was performed using the align function of PyMOL. Note that in the autoinhibited talin1, the R3, R8, and R11 domains are fully exposed to tensin3 TBS, whereas the tensin3 binding to R4 (highlighted by the red dashed circle) is interfered by the talin R1 domain.

The tensin3-binding regions in talin have been identified as binding sites for several other proteins, including actin (Gingras et al., 2008; Hemmings et al., 1996; Lee et al., 2004), caskin2 (Wang et al., 2024), RIAM, paxillin, and DLC1 (Chang et al., 2014; Lu et al., 2022; Zacharchenko et al., 2016), which rely on the three-dimensional folds of the rod domains being intact, and vinculin (Fillingham et al., 2005; Gingras et al., 2005), whose 11 binding sites (Bass et al., 1999; Gilmore et al., 1993; Gingras et al., 2005) are cryptic and require mechanical force to modulate their availability (del Rio et al., 2009; Papagrigoriou et al., 2004; Yao et al., 2016). While we have previously shown that talin (unless activated) does not bind vinculin in our MTS assay (Atherton et al., 2020), tensin3 readily binds talin in this assay. This observation is consistent with our AlphaFold3 model, which shows that the R3, R8, and R11 domains are accessible for tensin3 in the globular inactive talin conformation (Fig. S5). Talin activation may further lead to the unmasking of R4 to bind tensin3, which is supported by the observation that constitutively active talin (E1770A) (Goult et al., 2009) increases tensin3 coprecipitation compared with WT talin. This suggests that talin conformational changes, at least in part, modulate tensin3 binding (Fig. 7, H and I). However, the regulation of how the talin rod domains engage with their binding partners in time and space remains unclear. It is possible that the differential localization of adhesion proteins in cultured cells during adhesion maturation reflects the spatiotemporal competition, with subsets of proteins being recruited early (e.g., vinculin and paxillin) and tensin3 at later stages to cell–matrix adhesions.

The maturation of FAs to FBs involves actomyosin-dependent translocation of active α5β1 integrins toward the cell center (Pankov et al., 2000; Zamir et al., 2000). Integrins in FBs remain active even in the absence of actomyosin activity (Atherton et al., 2020; Zamir et al., 2000). Tensins have been proposed to mediate this activity by binding directly to integrins through their PTB domain (Georgiadou et al., 2017; McCleverty et al., 2007). The findings of the present study demonstrate that the integrin-binding PTB domain is not essential for either the formation of FBs or their maintenance in the absence of actomyosin forces (Fig. 5, J and K; and Fig. S3, H–K). Conversely, we show that the tensin3–talin interaction drives FB formation and is required to maintain α5β1 integrins in an activated state in FBs. While such events may provide a rationale for the sustained stability of FBs in the presence of actomyosin inhibitors (Atherton et al., 2022), this model is somewhat at odds with the low levels of talin1 observed in these structures. However, this phenomenon may be explained by the presence of multiple tensin-binding sites in talin (Figs. 1 and S1) and the enrichment of talin2 in FBs (Praekelt et al., 2012), which also binds tensin3 (Fig. 2 H) (Atherton et al., 2022).

It has become clear that molecular transitions occur during adhesion maturation and that differences in composition may be associated with changes in signaling capacity and function. It has recently been demonstrated that various adhesion proteins undergo LLPS, including LIMD1 (Wang et al., 2021), tensin1 (Dibus et al., 2025, *Preprint*; Lee et al., 2023), and paxillin (Liang et al., 2024). Here, we have shown that tensin3 undergoes LLPS (Fig. 6). Interestingly, the phase separation of adhesion proteins appears to be linked to mechanical cues within cells. For example, mechanically activated vinculin recruits LIMD1 to FAs, where it forms LLPS condensates (Wang et al., 2021). Inhibiting actomyosin contractility reduces FAs and LIMD1 localization, thereby limiting LIMD1 condensation at adhesion sites. Therefore, it appears that the vinculin-mediated recruitment is involved in the spatial organization of LIMD1 phase separation to regulate adhesion dynamics (Wang et al., 2021). For tensin3, which colocalizes with LIMD1 (Fig. 6 D), the retention to cell–matrix adhesion is regulated by talin. Without its talin-binding site, tensin3 still localizes to FAs and develops a high propensity to undergo LLPS (Fig. S4 D). These observations are exciting given that cells *in vivo* experience large variations in the mechanical properties. Such changes could dramatically alter cell behavior through modulating the formation and contents of LLPS condensates (Alberti and Hyman, 2021; Banani et al., 2017; Kumar et al., 2025).

There seem to be notable differences in the tensin3 condensates compared with other proteins. Paxillin condensates in cells appeared to remain tethered to the membrane, undergo high levels of tyrosine phosphorylation, and promote cell spreading (Liang et al., 2024). Unlike the reported paxillin condensates, tensin3 condensates neither were tyrosine-phosphorylated Fig. S4 K nor contained integrins (Fig. 7 E), kindlin2, or GIT1 (Fig. 8 A). Tensin3 also differs from tensin1, with the latter’s LLPS being regulated by serine/threonine phosphorylation (Dibus et al., 2025, *Preprint*) and cell cycle (Lee et al., 2023). Although tensin1 and tensin3 are structurally similar and can be recruited into the condensates formed by each other (Dibus et al., 2025, *Preprint*) (Fig. S4 I), they appear to compartmentalize different subsets of adhesion proteins. For example, talin, vinculin, or KANK is enriched in tensin3 condensates (Fig. 8 C) but almost absent from tensin1 condensates (Dibus et al., 2025, *Preprint*). Further study is required to elucidate the process by which these adhesion-driven LLPS condensates associate during the assembly and disassembly of integrin-mediated adhesions.

As with many other components undergoing LLPS (Banani et al., 2017; Wright and Dyson, 2015), the phase separation of tensin3 is mediated by its IDR (Fig. 6 C), which provides structural integrity for tensin3 condensates. As a scaffold component of these condensates (Banani et al., 2016; Banani et al., 2017), tensin3 selectively recruits a subset of client proteins into its condensates (Fig. 8 C). Talin, whose binding site is embedded in the IDR of tensin3, is a client in such condensates that is recruited via direct binding. Disrupting the tensin3–talin interaction also abolishes the localization of the talin-binding partner vinculin (Gingras et al., 2005) and KANK1/2 (Bouchet et al., 2016; Sun et al., 2016) to these condensates (Fig. 8 C), demonstrating that these proteins are secondary clients. The recruitment of DLC1 into tensin3 condensates is likely to be mediated by their direct interaction (Liao et al., 2006; Shih et al., 2015). Although little is known about GIGYF1, it contains multiple predicted IDRs. It was therefore intriguing to find it strongly enriched in tensin3 condensates and decorating filamentous structures, which are presumably microtubules (Fig. 8, A and B). While the precise mechanisms remain to be determined, our results suggest a scenario in which tensin3 condensates provide a platform that is influenced by the biophysical environment of cells. We therefore speculate that the mechanosensitively released tensin3 condensates could act as storage hubs that regulate the availability of adhesion proteins and as regulatory platforms that modulate signaling pathways, including actin organization (e.g., via DLC1 [Durkin et al., 2007; Shih et al., 2015]) and translational regulation (e.g., via GIGYF1 [Peter et al., 2017; Weber et al., 2020]).

**Figure 9.**
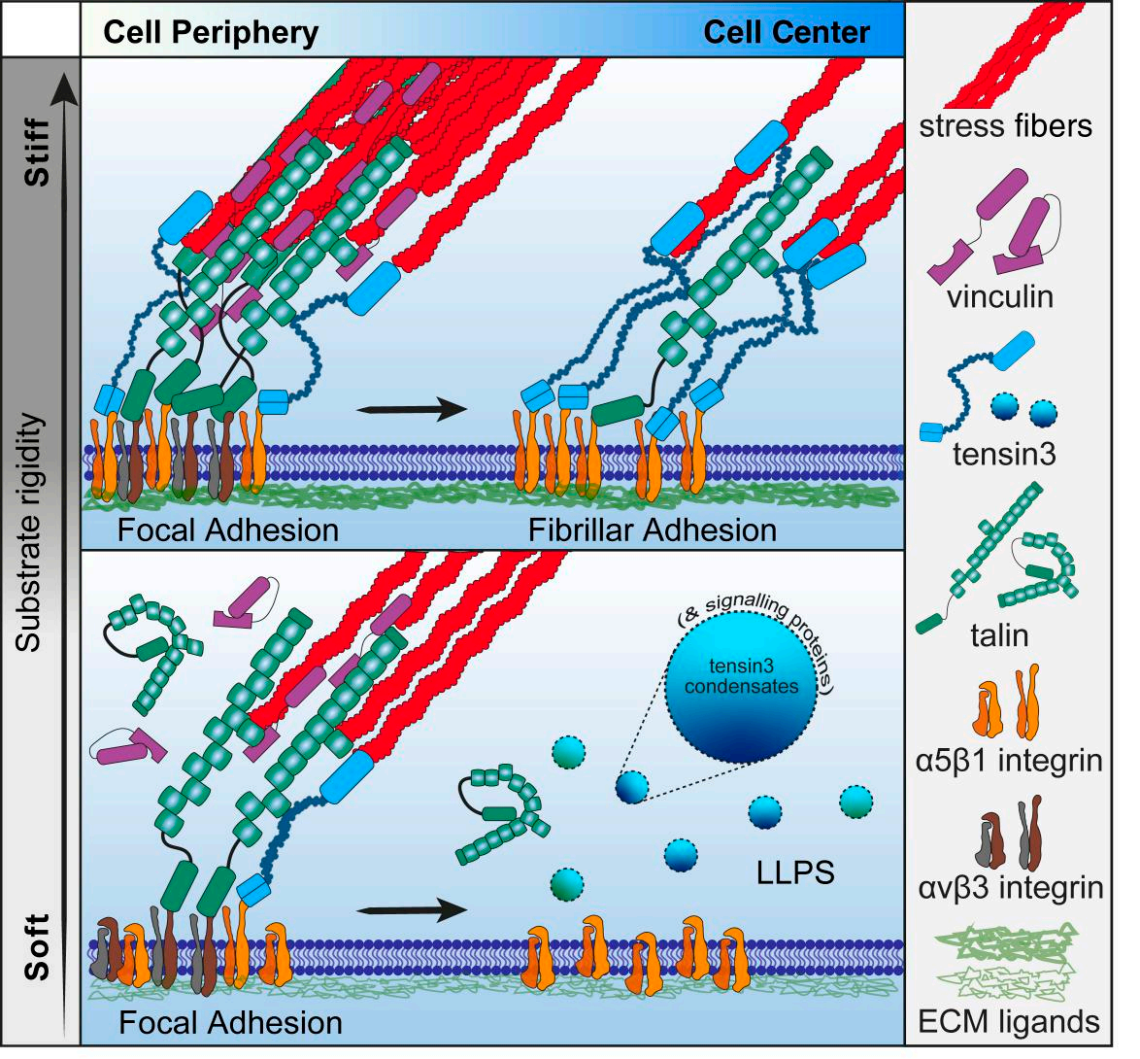
**Schematic model of tensin3 LLPS in response to rigidity sensing.** Schematic model of tensin3 recruitment to adhesions and its response to the surrounding mechanical environment. At the cell periphery, talin and other integrin activators (e.g., kindlin) activate integrins at the cell–ECM contact to form adhesion complexes. While talin and vinculin bind to actin filaments, actomyosin-mediated force induces the maturation of FAs with enrichment of α5β1 and αvβ3 integrins. The development of FAs into FBs depends on both substrate stiffness and the binding of tensin3 to talin. On stiff substrates, actomyosin-mediated forces stabilize talin in an active conformation that retains multiple tensin3 molecules during FA maturation into FBs. Tensin3 regulates integrin activity through its interactions with talin and integrins, which control the formation of force-independent stable FBs. On soft substrates, talin experiences lower forces and therefore exposes fewer binding sites for tensin3. Such reduced tensin3 retention in adhesions results in the formation of fewer FBs (Barber-Pérez et al., 2020) and increased tensin3 phase separation into biomolecular condensates. The tensin3 condensates could serve as storage compartments for adhesion proteins (e.g., talin) and initiate signaling (e.g., DLC1 and GIGYF1).

In summary, our findings shed light on the molecular mechanisms underlying the regulation of cell adhesion and suggest a potential link between tensin3 LLPS and cellular mechanotransduction. We propose a model that explains how talin cooperates with tensin3 during FB formation and regulates the mechanosensitive tensin3 LLPS (Fig. 9). At the cell periphery, integrins are linked to actomyosin by adhesion complexes containing active talin and vinculin, while actomyosin-mediated forces stabilize such complexes into FAs. Tensin3 localizes to the edge of mature FAs by interacting with talin and other proteins such as integrin (Atherton et al., 2022; McCleverty et al., 2007). As forces induce translocation of α5β1 integrins toward the cell center, a subset of adhesion proteins (e.g., vinculin) leave the complex, while more tensins enter and occupy the tensin-binding sites on talin, locking talin in an active conformation and maintaining integrin activity during FB formation. The mature FBs remain attached to the FN fibrils and keep essential adhesion signals active. This maturation process from FAs to FBs depends on both the tensin3–talin interaction and the stiffness of the substrate (Barber-Pérez et al., 2020). When cells encounter softer substrates, mechanosensory talin may be less efficient at retaining tensin3 in adhesions, causing the latter to undergo LLPS. These condensates can form a platform for readily available adhesion scaffolding and signaling proteins that support rapid cell responses to sudden environmental changes without the need to undergo more complex recycling systems.

## Materials and methods

### Cell culture and transfection

NIH3T3 mouse fibroblasts, HEK293T human epithelial cells, and U2OS human osteosarcoma cells were obtained from the American Type Culture Collection (ATCC).

U2OS TNS3KO cells were generated using CRISPR-Cas9 genome editing with a gRNA complex assembled from tensin3 crRNA (5′-AGU​CCG​CUC​CCG​CUC​AUA​G-3′, Sigma-Aldrich) and trRNA (IDT), and Cas9 nuclease V3 (IDT) (Atherton et al., 2022). HFFs were a gift from Patrick Caswell (University of Manchester, Manchester, UK). All cells were grown in Dulbecco’s modified Eagle’s medium (DMEM; Sigma-Aldrich) supplemented with 10% FBS (Gibco), 1% L-glutamine (Sigma-Aldrich), and 1% nonessential amino acids (Sigma-Aldrich). TLNKO cells (Atherton et al., 2015) were maintained in DMEM/F-12 (Gibco) supplemented with 10% FBS, 1% L-glutamine, 15 µM HEPES (Sigma-Aldrich), and 1% non-essential amino acids.

Transient transfections were performed using Lipofectamine LTX with Plus Reagent (Invitrogen) for NIH3T3 cells, Lipofectamine 2000 (Invitrogen) for U2OS cells and HFF cells, and jetPRIME (Polyplus) for TLNKO cells and HEK293T cells according to the manufacturer’s instructions. The same amount of plasmid cDNA was used for each transfection throughout the experiments to control the expression levels as much as possible. For live and fixed cell imaging, cells were plated on glass-bottom dishes (IBL) coated with 10 µg/ml bovine plasma FN (Sigma-Aldrich) diluted in PBS (Sigma-Aldrich).

### Antibodies and reagents

Cells were fixed in 4% paraformaldehyde (PFA, Sigma-Aldrich), which had been prewarmed to 37°C for 15 min before being washed three times with PBS. For immunofluorescence staining, cells were permeabilized with 0.5% Triton X-100 (Sigma-Aldrich) at room temperature for 5 min before being washed three times with PBS. Blebbistatin (Tocris Bioscience) was diluted in dimethyl sulfoxide (Sigma-Aldrich) and used at a final concentration of 50 μM. The following primary antibodies were diluted in 1% bovine serum albumin (BSA, Sigma-Aldrich): mouse anti-α5 integrin (SNAKA51, 1:100, NBP2-50146; Novus Biologicals), rat anti-β1 integrin (9EG7, 1:200; 553715; BD Biosciences), mouse anti-FN (IST-9, 1:100, ab6328; Abcam), rabbit anti-tensin3 (1:200, HPA055338; Atlas), mouse anti-EEA1 (1:500, 68065-1-IG; Proteintech), mouse anti-LAMP1 (1:200, ab320851; Abcam), mouse anti-paxillin (349, 1:400, mab3060; Sigma-Aldrich), rabbit anti-talin1 (1:200, 82856-4-RR; Proteintech), mouse anti-vinculin (hVin1, 1:500, V9264; Sigma-Aldrich), rabbit anti-KANK2 (1:100, HPA015643; Sigma-Aldrich), rabbit anti-kindlin2 (1:100, 11453-1-AP; Proteintech), rabbit anti-GIT1 (1:200, 26247-1-AP; Proteintech), mouse anti-phosphotyrosine (4G10, 1:200, 05-321X; Sigma-Aldrich), rabbit anti-G3BP1 (1:600, 13057-2-AP; Proteintech), rabbit anti-TDP43 (1:400, 10782-2-AP; Proteintech), and rabbit anti-GIGYF1 (1:400, ab121784; Abcam). Secondary antibodies, including Alexa Fluor Plus 488 goat anti-mouse (A32723; Invitrogen), Alexa Fluor Plus 594 goat anti-mouse (A32742; Invitrogen), Alexa Fluor Plus 488 goat anti-rabbit (A32731; Invitrogen), Alexa Fluor Plus 594 goat anti-rabbit (A32740; Invitrogen), and Alexa Fluor Plus 647 goat anti-rat (A48265; Invitrogen), were used at a dilution of 1:500. F-actin was visualized using Alexa Fluor Plus 405 phalloidin (A30104; Invitrogen) at a dilution of 1:500. Alexa Fluor Plus 405–conjugated WGA (W56132; Invitrogen) was used to visualize the plasma membrane and intracellular membranes. The cells were fixed and permeabilized, before being incubated in WGA (5 µg/ml) for 1 h.

### Protein expression and purification

Recombinant mouse talin1 R11R12 was expressed in a modified pET28a vector encoding an N-terminal decahistidine-tag followed by a 3C protease cleavage site and was synthesized by Twist Bioscience. Protein was expressed in T7 Express cells (NEB) and purified using a His-Trap column (Cytiva) with a linear gradient of imidazole (500 mM) and subsequently incubated with 3C protease (prepared in-house) overnight at 4°C. After a reverse pass of the column, the proteins were further purified using ion-exchange chromatography (QFF, Cytiva).

Recombinant mouse talin1 R3 and R7R8 domains were expressed using the pET-151/D-TOPO vector. The recombinant mouse talin R4 domain was expressed using the pET-28a(+)vectors. Both vectors include an N-terminal hexahistidine-tag followed by a tobacco etch virus protease cleavage site. Proteins were overexpressed in BL21 cells and purified via nickel-affinity chromatography with a single-step elution at 250 mM imidazole concentration. For NMR studies, uniformly ^15^N-labeled proteins were produced by growing cells in 2xM9 minimal medium supplemented with 1 g/Liter ^15^N-ammonium chloride (^15^NH_4_Cl) as the sole nitrogen source. Following purification, proteins were concentrated and buffer-exchanged using PD-10 desalting columns (Cytiva). Final protein concentrations were determined by measuring absorbance at 280 nm using a NanoDrop spectrophotometer (Thermo Fisher Scientific), based on calculated extinction coefficients.

### X-ray crystallography

Recombinant R11R12 protein was concentrated to 60 mg/ml in 20 mM Tris, pH 7.4, 50 mM NaCl, and 3 mM β-mercaptoethanol, and mixed with a fivefold molar excess of the tensin3 TBS peptide (aa 692–718). Crystals of the R11R12–tensin3 TBS complex were obtained in 0.9 M potassium sodium tartrate tetrahydrate, 0.05 M HEPES, pH 7.4, and 20% wt/vol glycerol at 294.15K using hanging drop (2 μl) vapor diffusion with a 1:1 protein–precipitate ratio. The crystals adopted a rod-like morphology and were vitrified in liquid nitrogen before data collection using mother liquor supplemented with 25% glycerol. Data were collected on beamline I04 (Diamond Light Source, Oxford) using a nonoverlapping 0.1° oscillation width over 360°. Data were scaled using XDS (Kabsch, 2010) and then merged using aimless (Evans, 2006) (data reduction statistics shown in Table S1) in space group P4_1_2_1_2/P4_3_2_1_2. The structure was solved by molecular replacement using the coordinates of R11 and R12 domains taken from the template structure 3DYJ in P4_3_2_1_2 with the PHENIX implementation of PHASER (McCoy et al., 2007) and refined with PHENIX using intensities (Adams et al., 2010). Following a single round of refinement, the complete electron density of the tensin3 peptides was visible on R11. The structure was refined using reference model restraints of the higher resolution experimentally phased model 3DYJ (1.85 Å) and optimized weights. Ordered solvents were picked both manually and by PHENIX/PDB-REDO (Joosten et al., 2014) on peaks above 3.0σ in the F_0_-F_C_ map. The asymmetric unit contained two R11R12 molecules and two tensin3 peptides. Chain C is strongest of the tensin3 peptides resolved with residues 692–715 fully resolved at 1σ in the 2F_0_-F_C_ map and simulated annealing composite omit map. The registry of the peptide is confirmed by the absolute position of the N terminus and a unique C-terminal ^708^ELDPTF^713^ motif that has captured ordered solvent. Chain D has diminished local resolution but was also assigned at 1σ in the 2F_0_-F_C_ map (refined maps and/SA omit maps are shown in Fig. S2, A and B). For R11R12, TLS groups were automatically determined by PHENIX and each peptide was considered a single group.

Structure factors and atomic coordinates were deposited to the PDB under the accession code: 9QN7, and the raw diffraction images were deposited to Zenodo with DOI: https://doi.org/10.5281/zenodo.15082702 (Zacharchenko, 2025). Data reduction and refinement statistics are shown in Table S1. The figures and maps were made by CCP4mg software (McNicholas et al., 2011).

### NMR spectroscopy

NMR spectra were acquired on Bruker Neo 700 MHz (for talin R3) or 800 MHz (for talin R4 and R7R8) spectrometers equipped with TCI cryoprobes. All experiments were performed at 298 K using 200 µM of uniformly ^15^N-labeled talin R3, R4, or R7R8 in 20 mM sodium phosphate buffer (pH 6.4) containing 50 mM NaCl, 0.1 mM tris(2-carboxyethyl)phosphine (TCEP), and 5% (vol/vol) D_2_O. For titration experiments, synthetic tensin3 TBS peptide (aa 692–718) was added at final concentrations of 50, 100, 200, 400, and 800 µM. NMR data were processed using TopSpin (Bruker) and analyzed with Collaborative Computational Project for NMR software AnalysisAssign (version 3.2). HSQC spectra were measured using a standard Bruker pulse sequence. HSQC signals of the free proteins were assigned using the available backbone chemical shift data from the Biological Magnetic Resonance Data Bank (BMRB entries; R3: 17332; R4: 18313). The signals of the bound state were assigned by following the chemical shift changes with the increased concentrations of the peptide. Residue-specific chemical shift differences were calculated using HSQC spectra in the presence of 400 µM tensin3 TBS peptide.

### Isothermal titration calorimetry

ITC experiments were performed using a MicroCal PEAQ-ITC automated instrument (Malvern Panalytical) at 25°C. Measurements of talin R3, R4, and R7R8 were conducted in 20 mM sodium phosphate and Tris buffer (pH 6.5) containing 50 mM NaCl and 0.5 mM TCEP, respectively. Data were analyzed using the one-site binding model implemented in MicroCal PEAQ-ITC analysis software (Malvern Panalytical).

### AlphaFold3 modeling

Models of R3, R4, R8, and R11 were performed using sequence data of their respective atomic coordinates (PDB: 2L7A, 2LQG, 2X0C, and 3DYJ, respectively) using the AlphaFold3 server (Abramson et al., 2024), and sequence of the tensin3 TBS peptide. Models with the highest confidence using the default output for the five structures were adopted. Confidence values of the atomic models and contact prediction plots are shown in Fig. S2, D, F and G.

### Plasmid preparation and site-directed mutagenesis

To generate the mitochondria targeting GFP-cBAK and mCh-cBAK vectors, the cBAK fragment (5′-TTG​CGT​AGA​GAC​CCC​ATC​CTG​ACC​GTA​ATG​GTG​ATT​TTT​GGT​GTG​GTT​CTG​TTG​GGC​CAA​TTC​GTG​GTA​CAC​AGA​TTC​TTC​AGA​TCA​TGA-3′) was cloned into the pEGFP-C1 (Clontech) and pmCherry-C1 (Clontech) vectors (Atherton et al., 2020). GFP-TLN1-cBAK was generated by inserting talin1 (*Mus musculus*) into the GFP-cBAK vector using restriction digestion (Atherton et al., 2020). GFP-TNS3 was a gift from David Critchley (University of Leicester, Leicester, UK). For the construction of mitochondrial targeting talin1 truncation constructs and TNS3-Cterm, PCR amplification was performed to obtain different cDNA fragments from GFP-TLN1 (*Mus musculus*) and mCh-TNS3 (*Homo sapiens*) using the primers listed in Table S3 and Q5 High-Fidelity 2× Master Mix (NEB). The mitochondrial targeting vector GFP-cBAK and pmCherry-C1 (Clontech) were linearized using XhoI (Thermo Fisher Scientific) and HindIII (Thermo Fisher Scientific) enzymes by incubation at 37°C for 60 min. Amplified fragments and digested vectors were run on 1% agarose gel (Sigma-Aldrich) mixed with SYBR Safe DNA stain (Thermo Fisher Scientific) together with a 100-kDa ladder (Bioline). Bands of the correct size were excised and purified using QIAquick Gel Extraction Kit (QIAGEN). DNA fragments and linearized vectors were assembled using HiFi DNA assembly (NEB) according to the manufacturer’s instructions.

To generate GFP-LIMD1 and mCh-LIMD1, full-length LIMD1 cDNA (*Homo sapiens*) was amplified from pTRIPZ-EGFP:LIMD1 (Ibar et al., 2018) using the primers listed in Table S3 and Q5 High-fidelity 2× master mix (NEB). The pTRIPZ-EGFP:LIMD1 construct (plasmid #108230; Addgene) was a gift from Kenneth Irvine (Rutgers University, Piscataway, NJ, USA). pEGFP-C1 (Clontech) and pmCherry-C1 (Clontech) were linearized using XhoI (Thermo Fisher Scientific) and HindIII (Thermo Fisher Scientific) enzymes by incubation at 37°C for 60 min. The amplified LIMD1 cDNA was assembled with the linearized pEGFP and pmCherry vector using the HiFi DNA assembly kit (NEB). The mCh-TLN2, mCh-TNS1, mCh-KANK1, and mCh-DLC1 were constructed by tagging the talin2 (*Homo sapiens*), tensin1 (*Homo sapiens*) (Clark et al., 2010), KANK1 (*Homo sapiens*) (Li et al., 2023), and DLC1 (*Homo sapiens*) cDNA in the C terminus of pmCherry-C1 (Clontech) via restriction digestion.

To generate GFP-tagged talin1 truncations, site-directed mutagenesis (SDM) was performed to introduce a stop codon before the mitochondrial targeting cBAK sequence using Q5 SDM Kit (NEB) according to the manufacturer’s instructions. Similar approaches were performed to generate mCh-TNS3-ΔPTB by introducing a stop codon before the PTB domain. All point mutations in talin1 and tensin3 were introduced by SDM. All primer information used for SDM is listed in Table S4.

### Mitochondrial isolation

Mitochondrial isolation from HEK293T cells was performed after 24 h of transient expression using a combined method of Qproteome Mitochondria Isolation Kit (QIAGEN) and MACS Mitochondria Isolation Kit (Miltenyi). Cells were collected in ice-cold PBS and aliquoted for the preparation of the whole-cell lysate (wcl) fraction (30%) and the mitochondrial fraction (70%). Cell lysis and homogenization of the mitochondrial fraction were performed according to the manufacturer’s instructions (QIAGEN). Magnetic anti-TOM22 microbeads (Miltenyi) were used to label mitochondria in the homogenized mitochondrial fraction for 1 h at 4°C. The labeled mitochondria were separated from a magnetic column before elution with ice-cold separation buffer (Miltenyi). The eluted mitochondria were washed and centrifuged according to the Miltenyi’s instructions. RIPA buffer (ChromoTek) was used for protein extraction from the wcl and purified mitochondria. Samples were stored at −80°C prior to western blotting.

### Western blot

Samples were mixed with sample buffer (4X, Invitrogen) supplemented with reducing agent (10X, Invitrogen). Samples were heated at 95°C for 5 min before loading onto 4–12% SDS-PAGE gels (Invitrogen). MOPS SDS running buffer (Invitrogen) supplemented with antioxidants (1:400, Invitrogen) was used. The gel was transferred to a 0.45 μm nitrocellulose membrane (Cytiva) blocked for 1 h in 5% skimmed milk (Sigma-Aldrich) in PBS/Tween-20 (0.1%, Sigma-Aldrich). The membrane was probed with anti-GFP (1:10,000, ab183734; Abcam), anti-mCherry (1:3,000, 1C51, ab125096; Abcam), anti-VDAC1 (1:1,500, ab15895; Abcam), and anti-α-tubulin (1:1,500, DM1α, T6199; Sigma-Aldrich), respectively, in 5% milk (PBS/Tween). Signals were detected using goat anti-mouse IgG conjugated to IRDye 680RD (1:15,000, ab216776; Abcam) and goat anti-rabbit IgG conjugated to IRDye 800CW (1:15000, ab216773; Abcam) secondary antibodies. An Odyssey CLx imaging system (LI-CO Biosciences) was used for signal visualization. Western blot analysis and quantification were performed using ImageJ software.

### Flow cytometry analysis

To measure the level of β1 integrin activation, TLNKO cells were transfected in a 6-well plate for flow cytometry analysis. Cells were collected, washed, and resuspended in ice-cold staining buffer (1% BSA in PBS) before incubation with 9EG7 antibody (diluted 1:200 in staining buffer) for 1 h on ice. The cells were then washed three times with staining buffer. Alexa Fluor Plus 647 goat anti-rat (1:500 dilution in staining buffer, Invitrogen) secondary antibody was used to incubate the cells for 45 min on ice, before being washed twice with staining buffer and once with PBS. The cells were then fixed with 4% PFA for 15 min before a final wash with PBS. Flow cytometry analysis was performed using a Fortessa system (BD Biosciences) and FlowJo software.

### Soft/stiff PDMS substrates and polyacrylamide (PAA) hydrogel preparation

Imaging dishes equipped with 40 µm-thick PDMS substrates (1.5 and 28 kPa) were purchased from ibidi. PDMS dishes were coated with 30 μg/ml FN for 1 h, followed by three washes with PBS. Dishes were incubated with DMEM for 10 min to equilibrate before cells were plated and incubated overnight at 37°C.

Prior to the preparation of PAA hydrogels (5 and 50 kPa), the glass-bottom dishes (IBL) were cleaned with 0.1 M of NaOH for 5 min, then treated with (3-aminopropyl)triethoxysilane (APES, Sigma-Aldrich) for 4 min to perform aminosalinization. The APES was diluted with 4 ml of PBS and then removed from the dishes by excessive washing with water. The dishes were then incubated with 0.5% glutaraldehyde (diluted in PBS) for 30 min at room temperature, before being washed with water and being placed in 70% ethanol overnight. On the next day, PAA gels were prepared with ProtoGel 30% (37.5:1 ratio of acrylamide to bisacrylamide solution, National Diagnostics) diluted in PBS (ratio was adjusted for 5 and 50 kPa), 10% ammonium persulfate (Sigma-Aldrich), and 0.01% (vol/vol) tetramethylethylenediamine (Sigma-Aldrich). Meanwhile, round coverslips (Marienfeld) were coated with 50 μg/ml FN (Sigma-Aldrich) diluted in PBS for 1 h. To prepare a thin layer of gel, 10 μl of prepared PAA gel was added in the middle of the air-dried glass-bottom dish, and the coverslip was placed on the top with the FN-coated side toward the gel (Atherton et al., 2022). The dishes were incubated at 37°C for 30 min to allow the transfer of FN from the coverslip into the gel. The dishes were then incubated with PBS for 30 min before the coverslip was removed. Finally, the dishes were washed three times with PBS before being plated with cells and incubated overnight at 37°C.

### Microscopy

#### Fixed-cell imaging

Imaging of fixed cells in PBS in glass-bottom dishes was carried out at room temperature. For all fixed cells except the mCh-TNS3-expressing NIH3T3 cells on the PDMS dishes, imaging was performed using an Olympus IX83 inverted microscope equipped with a 60×/1.42 PlanApoN oil objective and 20×/0.85 UPlan S Apo oil objective lenses. Metamorph software (version v7.10, Molecular Devices) was used to control the microscopy system. The samples were illuminated with LEDs (UV/cyan/green-yellow/red, Lumencor) for fluorescence excitation: UV (395 nm) for Alexa Fluor Plus 405; cyan (470 nm) for EGFP and Alexa Fluor Plus 488; green-yellow (550 nm) for mCherry and Alexa Fluor Plus 594; red (640 nm) for Alexa Fluor Plus 647. A Sedat band-pass filter set (DAPI/FITC/TRITC/Cy5, Chroma, 89000) was used to collect blue (DAPI, 433/24 nm), green (FITC, 520/35 nm), yellow/orange (TRIC, 600/37 nm), and far-red emission (Cy5, 680/42 nm). The images were collected using a Retiga R6 CCD camera (QImaging) without pixel binning. Z-stack images were collected with a Z optical spacing of 0.2 μm. For each quantitative experiment, the cells were imaged with the same exposure time to allow comparison of fluorescence intensities in the different conditions.

For NIH3T3 cells expressing mCh-TNS3 in PBS on PDMS dishes, images were collected using a Leica TCS SP8 AOBS upright confocal equipped with an HCX Apo 63×/0.90 immersion objective at room temperature. A 594-nm laser line was used to excite mCherry. Images were collected with hybrid detectors with detection mirror setting of mCherry 602–665 nm. LAS X software (version v3.5.1.18803, Leica) was used to operate the microscopy system.

#### Live-cell imaging

NIH3T3 cells were transfected in 12-well plates and plated the next day on FN-coated glass-bottom dishes (IBL). One hour before imaging, the medium was replaced with prewarmed DMEM medium supplemented with 10% FBS (Gibco) and 1% L-glutamine (Sigma-Aldrich). Imaging was carried out at 37°C with supplement of 5% CO_2_. Images were acquired using a spinning disk confocal (Yokogawa) on a Zeiss Axio Observer Z1 microscope equipped with a 60×/1.40 Plan-Apochromat objective, an Evolve EMCCD camera (Photometrics), and a motorized XYZ stage (ASI). A 488-nm laser line was used to excite EGFP. The 488-nm laser was controlled by an acousto-optic tunable filter through the laser stack (Intelligent Imaging Innovations, 3I). The microscope system was controlled using Slidebook software (version 6.0.3, 3I).

#### Fluorescence recovery after photobleaching

Transfected NIH3T3 cells were incubated in prewarmed DMEM medium at 37°C for 1 h to equilibrate prior to imaging. FRAP experiments were performed using a spinning disk confocal microscope as described above. Slidebook software (version 6.0.3, 3I) was used to set up the experiment. Regions of interest (ROIs) were manually adjusted to the shape of adhesion. A 488-nm laser was used at 100% power to bleach one to three adhesion ROIs per cell. Three prebleach images were acquired, followed by one image every 10 s for 3 min after bleach. Time-lapse images were analyzed using FIJI/ImageJ software (Schindelin et al., 2012) to obtain the intensity values of three control unbleached control ROIs and bleached ROIs over time. Values were background-subtracted, and measurements were corrected with the control values of the unbleached ROIs to compensate for any overall fluorescence loss. Intensity values were then normalized to the first postbleach value; normalized data were fitted to a one-phase association model Y=Y0+(Plateau-Y0)*(1-exp(-K*x)) using GraphPad Prism 10 software. Coefficients of the curve fit were extracted and transformed to generate the mobile fraction and halftime of recovery.

### Analysis of adhesions, FN fibrils, and phase-separated condensates

Analysis of cell adhesion sites, FN fibrils, and LLPS condensates was performed using FIJI/ImageJ (version 1.54f) software (Schindelin et al., 2012). The quantification of adhesion was performed on transfected cells that exhibited low expression levels. Prior to adhesion quantification, the images were background-subtracted using a rolling ball algorithm, followed by thresholding of adhesion sites and Analyse Particles functions in FIJI/ImageJ to quantify adhesions. Adhesion sites with sizes between 0.4 and 10 μm^2^ were counted, except for adhesions in blebbistatin-treated cells, which were counted with sizes between 0.2 and 4 μm^2^. To perform the distance measurement of adhesion sites, an ROI was drawn around the cell periphery, which was used to create a Euclidean distance map (EDM) using the Distance Map function in FIJI/ImageJ. The distance values were normalized to the maximum value within each cell and multiplied by 100 (Atherton et al., 2022). Adhesion sites were thresholded as above and masked before being applied to the EDM to generate the mean distance value of each adhesion site.

To analyze the FN fibrils, the images were background-subtracted (rolling ball), before 40 × 40 μm square boxes were applied for particle analysis to count FN fibrils larger than 0.2 μm^2^. The FN coverage was calculated by dividing the area covered by FN fibrils by the total area of the box (1,600 μm^2^).

To establish a correlation between tensin3 expression and condensation formation, a maximum intensity Z-projection was performed on Z-stack images of NIH3T3 cells (step depth 0.2 μm). The integrated fluorescence intensity was measured by normalizing the mean pixel intensity of each cell to the average of all values in cells expressing mCh-TNS3. To measure the condensate coverage in NIH3T3 cells, the images were background-subtracted (rolling ball) and then thresholded. This was followed by the Analyse Particles function in FIJI/ImageJ to select round condensate structures (circularity 0.7–1.0) (Bussi et al., 2023; Dumelie et al., 2024) with a size larger than 0.1 μm^2^. The same settings were applied to quantify the number of condensates in NIH3T3 and HFF cells.

The percentage of NIH3T3 cells transfected with mCh-TNS3 that form phase-separated condensates was quantified manually in images (610 × 499 μm) acquired with a 20× objective. To quantify the mean radius (R) of the condensates, the area covered by the condensates was divided by the number of condensates for each cell to obtain the mean size (A) of the condensates, followed by the formula of R = √(A/3.14).

### Graphs and statistical analysis

All graphs and statistical analyses were generated and performed using GraphPad Prism 10 software (version 10.4.0). Where appropriate, statistical significance between two single groups was tested using an unpaired *t* test, Welch’s *t* test, Mann–Whitney test, or analysis of covariance. Significance between more than two groups was tested using repeated-measures one-way ANOVA with Dunnett’s multiple comparisons, ordinary one-way ANOVA with Tukey’s multiple comparisons, or Kruskal–Wallis test with Dunn’s multiple comparisons, as appropriate. The results that were considered to be statistically significant are shown: *P < 0.05; **P < 0.01; ***P < 0.001; ****P < 0.0001. Data distribution was tested for normality using the D’Agostino & Pearson omnibus K2 test. A P value >0.05 was used to determine the normality.

### Online supplemental material

Fig. S1 (related to Figs. 1 and 2) shows the tensin3 binding to talin R3, R4, R7R8, and R11 domains, and the L702E mutation disrupts the tensin3–talin interaction. Fig. S2 (related to Fig. 3) shows the structural characterization of the multidomain tensin3–talin interaction. Fig. S3 (related to Fig. 5) shows tensin3 contributes to integrin activity through its PTB domain and through its interaction with talin. Fig. S4 (related to Figs. 6, 7, and 8) shows talin regulates tensin3 LLPS in cells that compartmentalize adhesion proteins and signaling molecules. Fig. S5 shows the R3, R8, and R11 domains are accessible for tensin3 in the globular inactive talin conformation. Video 1 (related to Fig. 6 F) shows the formation of a GFP-TNS3 condensate at adhesion sites and release into the cytoplasm. Video 2 (related to Fig. 6 G) shows the fusion of two GFP-TNS3 condensates upon contact. Table S1 shows the data reduction and refinement statistics for the crystal structure of the talin1 R11R12–tensin3 TBS complex. Table S2 shows the list of point mutations tested in the talin R3 and R4 domains. Table S3 shows the list of primers used for cDNA amplification. Table S4 shows the list of primers used for SDM.

## Supplementary Material

Table S1 shows data reduction and refinement statistics.

Table S2 shows list of point mutations tested for talin R3 and R4 domains.

Table S3 shows list of primers used for cDNA amplification.

Table S4 shows list of primers used for SDM.

SourceData F1 is the source file for Fig. 1.

SourceData F2 is the source file for Fig. 2.

SourceData F3 is the source file for Fig. 3.

SourceData F7 is the source file for Fig. 7.

SourceData FS1 is the source file for Fig. S1.

## Data Availability

Atomic coordinates and structure factors of the talin1 R11R12–tensin3 TBS complex have been deposited to the PDB with the accession code 9QN7. The raw diffraction images were deposited to Zenodo with DOIs: https://doi.org/10.5281/zenodo.15082702 for R11R12–TBS; and https://doi.org/10.5281/zenodo.17286977 for R7R8–TBS. The data related to testing the point mutations listed in Table S2 may be requested from the authors. All the other data necessary for evaluating the conclusions in the paper are included in the paper and the supplementary materials. Source western blot images for Fig. 1 E; Fig. 2 F; Fig. 3, B and E; Fig. 7 H; and Fig. S1, A–C are available in the online supplemental material.
