# Supplementary material for: Talin–tensin3 interactions regulate fibrillar adhesion formation and tensin3 phase separation
Source: J Cell Biol. 2025 Nov 21;225(1):e202503155. doi: 10.1083/jcb.202503155 (PMC12637021; doi:10.1083/jcb.202503155)
Supplement: Table S1 — shows data reduction and refinement statistics. [file jcb_202503155_tables1.docx]

|  | **R11R12-tensin3 TBS** |
| --- | --- |
| **Beamline** | **I04-Diamond** |
| Wavelength (Å) | 0.9537 |
| Resolution range (Å) | 86.58-2.76 (2.89-2.76) |
| Space group | P4_3_2_1_2 |
| *a, b, c* (Å) | 84.94, 84.94, 346.33 |
| *α, β, γ (^o^)* | 90.00, 90.00, 90.00 |
| Unique reflections | 33905 (4373) |
| Completeness (%) | 99.9 (100) |
| Multiplicity | 26.2 (27.7) |
| Wilson B-factor (Å^2^) | 64.3 |
| CC_1/2_ | 0.999 (0.483) |
| <I/σ(i)> | 10.6 (1.4) |
| R_merge_ (I) | 0.441 (3.087) |
| R_p.i.m_ | 0.087 (0.590) |
| **Refinement** | |
| Reflections used in refinement | 33801 |
| R_work_/R_free (%)_ | 25.70/29.58 |
| No of atoms | 5102 |
| Macromolecule | 5081 |
| Solvent | 21 |
| RMSD bonds (Å) | 0.005 |
| RMSD angles (^o^) | 0.950 |
| **Ramachandran** | |
| Favoured (%) | 97.49 |
| Allowed (%) | 1.92 |
| **Average B-factor all atoms (Å^2^)** | 89 |
| **Whole-Chain B-factors (Å^2^)** |  |
| Chain A | 84.03 |
| Chain B | 90.13 |
| Chain C | 102.09 |
| Chain D | 153.21 |

**Table S1.** **Data reduction and refinement statistics.**

*R_free_* was calculated using 5.01% of data isolated from the refinement for cross-validation. Overall values are shown, with the highest resolution shell in parenthesis.
