# Supplementary material for: Talin–tensin3 interactions regulate fibrillar adhesion formation and tensin3 phase separation
Source: J Cell Biol. 2025 Nov 21;225(1):e202503155. doi: 10.1083/jcb.202503155 (PMC12637021; doi:10.1083/jcb.202503155)
Supplement: Table S2 — shows list of point mutations tested for talin R3 and R4 domains. [file jcb_202503155_tables2.docx]

|  | **GFP-R1R3-cBAK** | **GFP-R4R6-cBAK** |
| --- | --- | --- |
| **Mutation(s)** | R827E | K999E |
|  | K841E | K1025E |
|  | K861E | K999E+K1025E |
|  | K875E | K999E+K1035E |
|  | R827E+K875E | K999E+K1025E+K1035E |
|  | K841E+K861E | K999E+K1005E+K1025E+K1035E |
|  | R827E+K841E+K875E | K1068E |
|  | R827E+K841E+K861E+K875E | K1096E |
|  |  | K1190E |

**Table S2. List of point mutations tested for talin R3 and R4 domains.**

Note that the point mutations in talin R3 (left column) and R4 (right column) were introduced on mitochondria targeting talin truncation construct GFP-R1R3-cBAK or GFP-R4R6-cBAK, respectively, and then tested for colocalisation with mCh-TNS3-IDR.
