# Supplementary material for: Talin–tensin3 interactions regulate fibrillar adhesion formation and tensin3 phase separation
Source: J Cell Biol. 2025 Nov 21;225(1):e202503155. doi: 10.1083/jcb.202503155 (PMC12637021; doi:10.1083/jcb.202503155)
Supplement: Table S3 — shows list of primers used for cDNA amplification. [file jcb_202503155_tables3.docx]

| **Sequence** | **Primers for PCR Amplification** | | **Construct** |
| --- | --- | --- | --- |
| **Talin1 R1** | Forward | actctcggcatggacgagctgtacaagtccggccggactcagatccagcagtacaaccgagtggggaaag | **GFP-R1-cBAK** |
|  | Reverse | accattacggtcaggatggggtctctacgcaagaattcgaaaatttgctgcaacagctccccactgg |  |
| **Talin1 R1R2** | Forward | actctcggcatggacgagctgtacaagtccggccggactcagatccagcagtacaaccgagtggggaaag | **GFP-R1R2-cBAK** |
|  | Reverse | accattacggtcaggatggggtctctacgcaagaattcgaacttcacgtgctgcagcagctcattgag |  |
| **Talin1 R1R3** | Forward | actctcggcatggacgagctgtacaagtccggccggactcagatccagcagtacaaccgagtggggaaag | **GFP-R1R3-cBAK** |
|  | Reverse | accattacggtcaggatggggtctctacgcaagaattcgaacttcttgatggcgttctgcgcagctgcattgg |  |
| **Talin1 R4R5** | Forward | cggcatggacgagctgtacaagtccggccggactcagatcttggtgcagcgcctggagcatgcagcc | **GFP-R4R5-cBAK** |
|  | Reverse | accattacggtcaggatggggtctctacgcaagaattcgaattggccaggcaggcagctgacacagc |  |
| **Talin1 R4R6** | Forward | cggcatggacgagctgtacaagtccggccggactcagatcttggtgcagcgcctggagcatgcagcc | **GFP-R4R6-cBAK** |
|  | Reverse | accattacggtcaggatggggtctctacgcaagaattcgaatgcctgctgggtgcacatggtgatgag |  |
| **Talin1 R5R6** | Forward | cggcatggacgagctgtacaagtccggccggactcagatcggacctttggagatggattctgca | **GFP-R5R6-cBAK** |
|  | Reverse | accattacggtcaggatggggtctctacgcaagaattcgaatgcctgctgggtgcacatggtgatgag |  |
| **Talin1 R7R8** | Forward | actctcggcatggacgagctgtacaagtccggccggactcagatcgcacctggccagaaggagtgtgacaat | **GFP-R7R8-cBAK** |
|  | Reverse | accattacggtcaggatggggtctctacgcaagaattcgaagtccctcatgcttgtaataagtttcttga |  |
| **Talin1 R9R10** | Forward | cggcatggacgagctgtacaagtccggccggactcagatcgccccagggcagctggagtgtgaga | **GFP-R9R10-cBAK** |
|  | Reverse | accattacggtcaggatggggtctctacgcaagaattcgaaacgattcccagcctggagtgcagccag |  |
| **Talin1 R11DD** | Forward | actctcggcatggacgagctgtacaagtccggccggactcagatcggtacccaggcctgcattacagcagcc | **GFP-R11DD-cBAK** |
|  | Reverse | accattacggtcaggatggggtctctacgcaagaattcgaagtgctcgtctcgaagctctgaagg |  |
| **Talin1 R12DD** | Forward | cggcatggacgagctgtacaagtccggccggactcagatcgatgaggccaccaaaggcacacgggc | **GFP-R12DD-cBAK** |
|  | Reverse | accattacggtcaggatggggtctctacgcaagaattcgaagtgctcgtctcgaagctctgaagg |  |
| **Tensin3 C-termimus** | Forward | cggcatggacgagctgtacaagtccggccggactcagatcatgttctccagcccgcacagcgggagca | **mCh-TNS3-Cterm** |
|  | Reverse | accattacggtcaggatggggtctctacgcaagaattcgaagaccttctttggtgaaccaatcatgacct |  |
| **Tensin3 IDR deletion** | Forward | cggcatggacgagctgtacaagtccggactcagatccatgagagagggccatgggctgga | **mCh-TNS3-ΔIDR** |
|  | Reverse | atcacctggactatcatcgacagggccctgcgtgtgtagcacttc |  |
|  | Forward | cagggccctgtcgatgatagtccaggtgataaacttgtgatcgtg |  |
|  | Reverse | accattacggtcaggatggggtctctacgcaagaattcgaagaccttctttggtgaaccaatcatgacct |  |
| **LIMD1** | Forward | cggcatggacgagctgtacaagtccggactcagatccatggataagtatgacgacctgggcctggag | **GFP-LIMD1/mCh-LIMD1** |
|  | Reverse | ggatcccgggcccgcggtaccgtcgactgcagaattcgaatcagttatctagactgaagtggtgctggtgaag |  |

**Table S3. List of primers used for cDNA amplification.**
