# Supplementary material for: Talin–tensin3 interactions regulate fibrillar adhesion formation and tensin3 phase separation
Source: J Cell Biol. 2025 Nov 21;225(1):e202503155. doi: 10.1083/jcb.202503155 (PMC12637021; doi:10.1083/jcb.202503155)
Supplement: Table S4 — shows list of primers used for SDM. [file jcb_202503155_tables4.docx]

| **Primers for Site-Directed Mutagenesis** | | |
| --- | --- | --- |
| **Talin1 R1R2 stop codon** | Forward | CGTGAAGTTCtAATTCTTGCGTAGAG |
|  | Reverse | TGCTGCAGCAGCTCATTG |
| **Talin1 R1R3 stop codon** | Forward | CAAGAAGTTCtAATTCTTGCGTAGAGACCCC |
|  | Reverse | ATGGCGTTCTGCGCAGCT |
| **Talin1 R4R6 stop codon** | Forward | GCAGGCATTCtAATTCTTGCGTAG |
|  | Reverse | TGGGTGCACATGGTGATG |
| **Talin1 R5R6 stop codon** | Forward | GCAGGCATTCtAATTCTTGCGTAG |
|  | Reverse | TGGGTGCACATGGTGATG |
| **Talin1 R7R8 stop codon** | Forward | GAGGGACTTCtAATTCTTGCG |
|  | Reverse | ATGCTTGTAATAAGTTTCTTG |
| **Talin1 R9R10 stop codon** | Forward | GAATCGTTTCtAATTCTTGCGTAGAGACCCC |
|  | Reverse | CCAGCCTGGAGTGCAGCC |
| **Talin1 end stop codon** | Forward | CGAGCACTTCtAATTCTTGCG |
|  | Reverse | TCTCGAAGCTCTGAAGGC |
| **Tensin3 L702E** | Forward | CATCGAGCAGgaaAACAGGCTGATCC |
|  | Reverse | GACTGGTCGATGTCCAGG |
| **Tensin3 I706E** | Forward | CAACAGGCTGgaaCTGGAGCTGGATC |
|  | Reverse | AGCTGCTCGATGGACTGG |
| **Tensin3 L707E** | Forward | CAGGCTGATCgagGAGCTGGATC |
|  | Reverse | TTGAGCTGCTCGATGGAC |
| **Talin1 K1500E** | Forward | TATTGTAGCCgAACACACATCTG |
|  | Reverse | GTGGCTGCAGATAACACC |
| **Talin1 R1510E** | Forward | TAACAGCTGTgaaCTGGCTTCCG |
|  | Reverse | CACAATGCAGATGTGTGTTTG |
| **Talin1 K2024E** | Forward | AAAGACAGCAgAGGTTCTTGTGG |
|  | Reverse | AAGATACCCTCCCGGTGG |
| **Talin1 K2031E** | Forward | GGAGGACACCgAGGTCCTAGT |
|  | Reverse | ACAAGAACCTcTGCTGTCTTTAAG |
| **Tensin3 PTB stop codon** | Forward | GCAGGGGGCAtagTGCAATGTGTGG |
|  | Reverse | TTCAACAGCTCAGCTGCT |

Table S4. List of primers used for site-directed mutagenesis
