# Supplementary figures and images for: Talin–tensin3 interactions regulate fibrillar adhesion formation and tensin3 phase separation
Source: J Cell Biol. 2025 Nov 21;225(1):e202503155. doi: 10.1083/jcb.202503155 (PMC12637021; doi:10.1083/jcb.202503155)

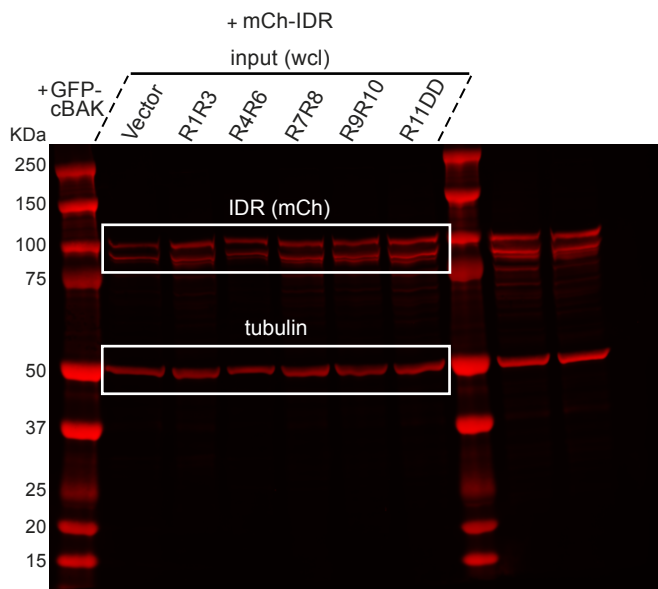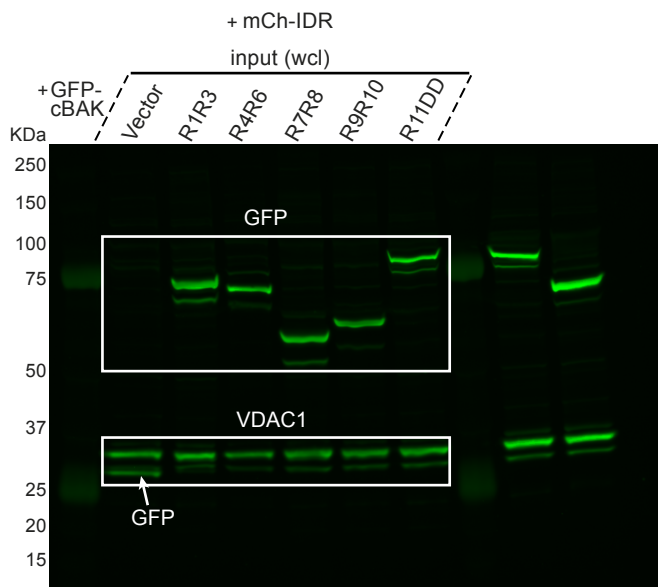

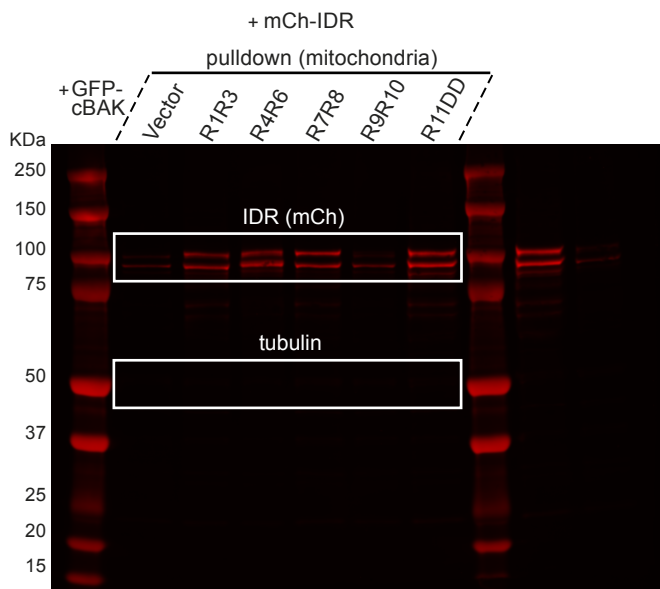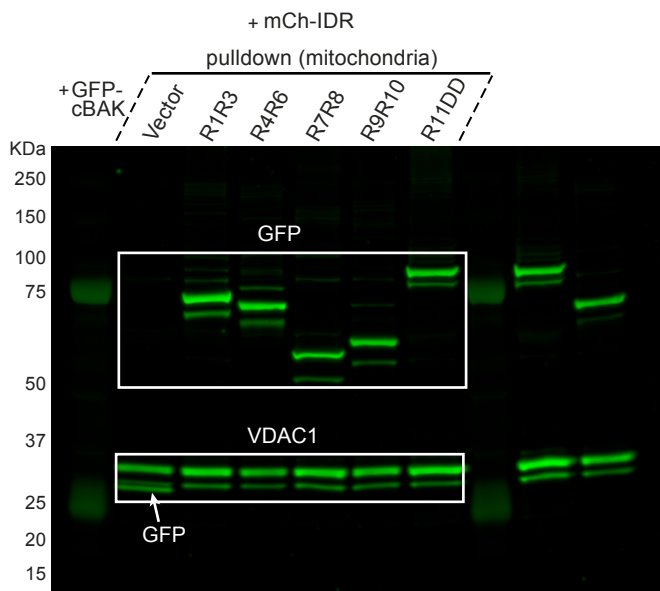

Supplement: SourceData F1 — is the source file for Fig. 1. [file jcb_202503155_sourcedataf1.pdf]

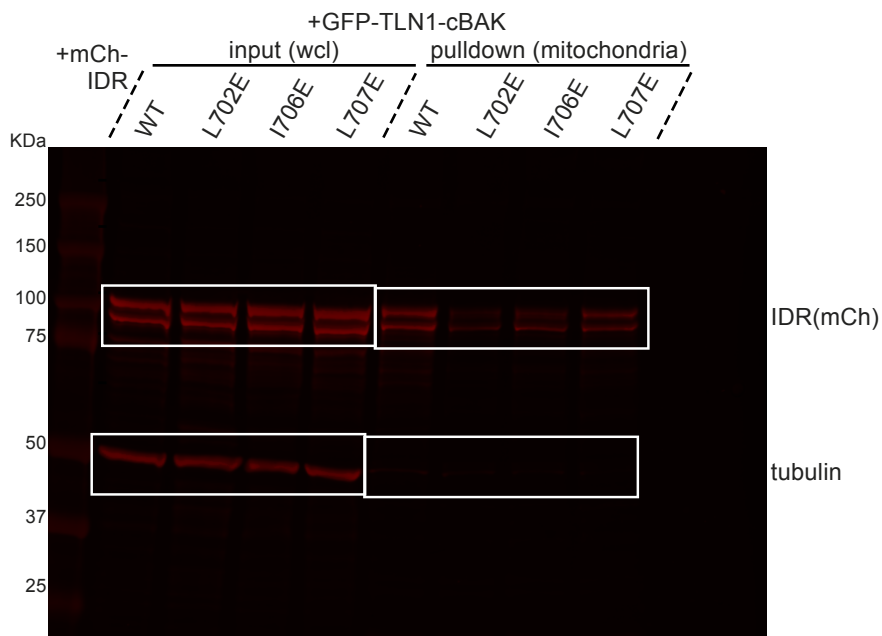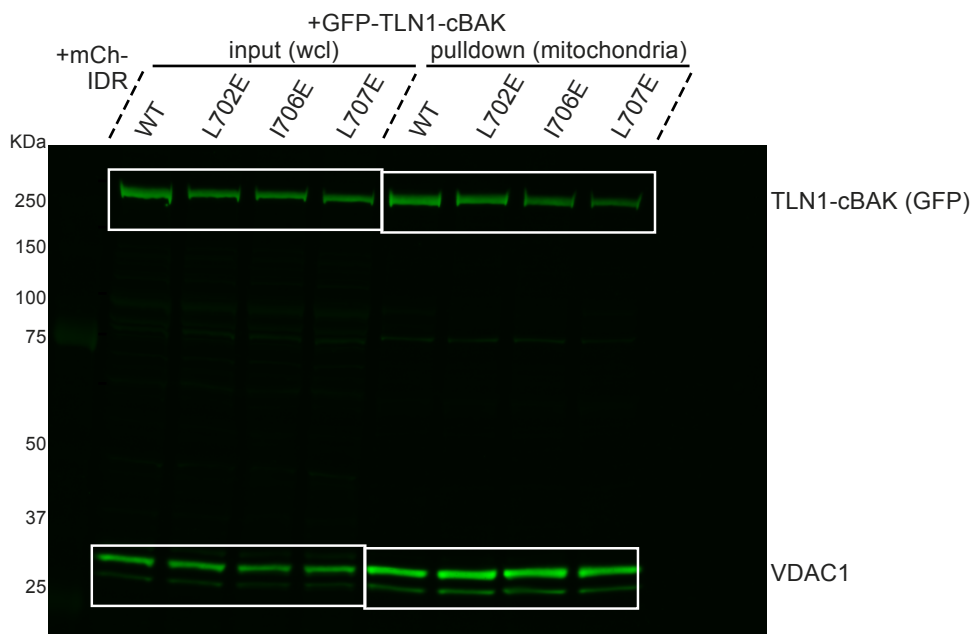

Supplement: SourceData F2 — is the source file for Fig. 2. [file jcb_202503155_sourcedataf2.pdf]

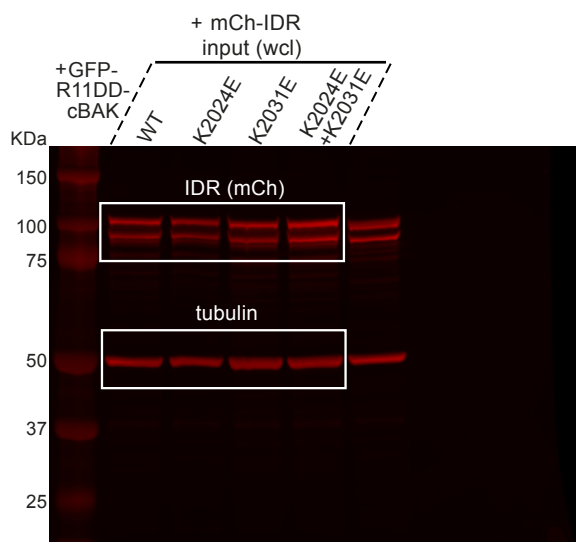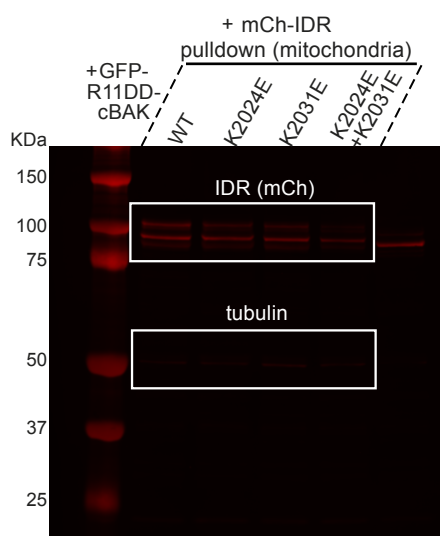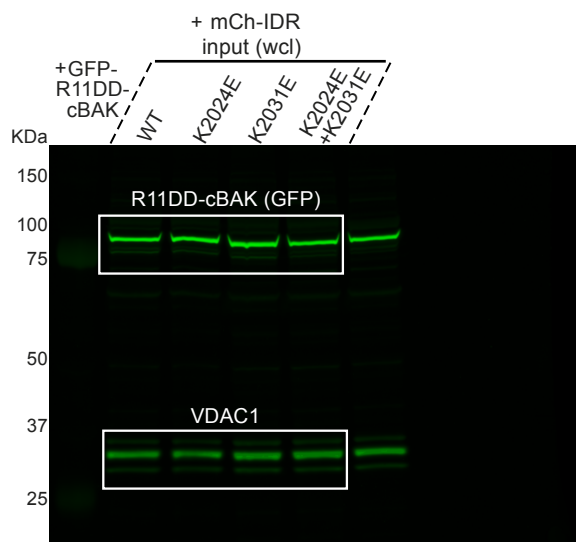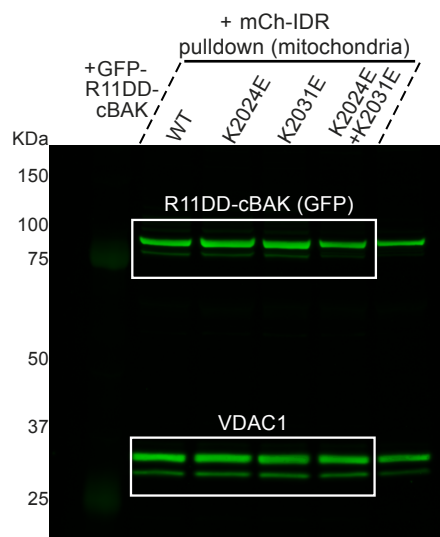

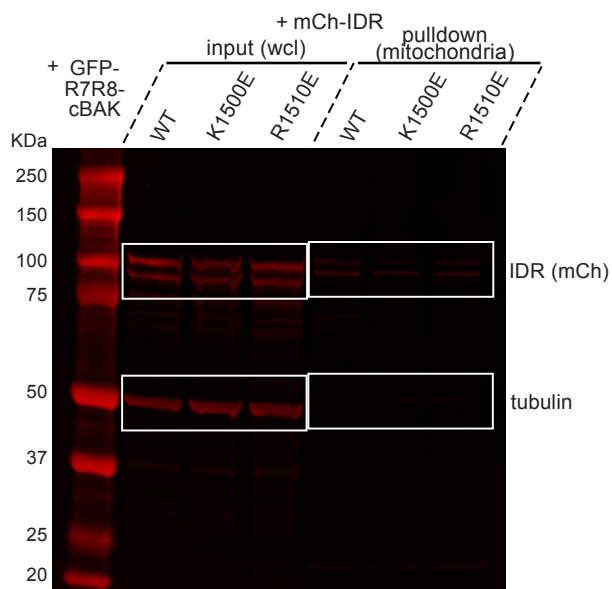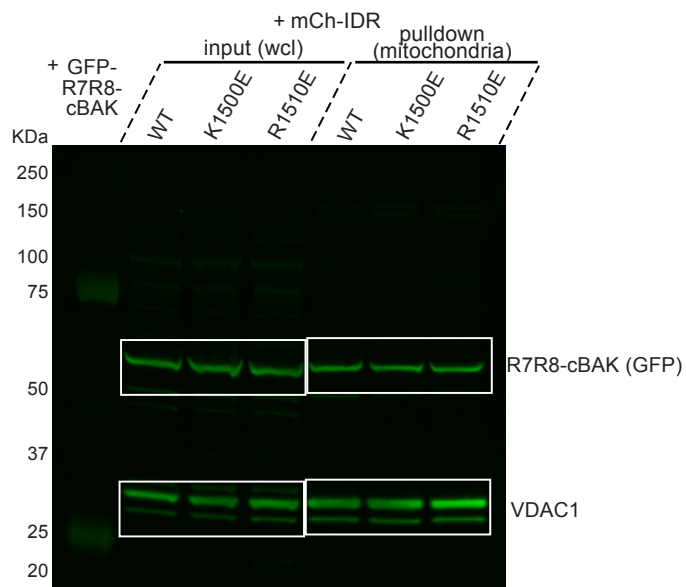

Supplement: SourceData F3 — is the source file for Fig. 3. [file jcb_202503155_sourcedataf3.pdf]

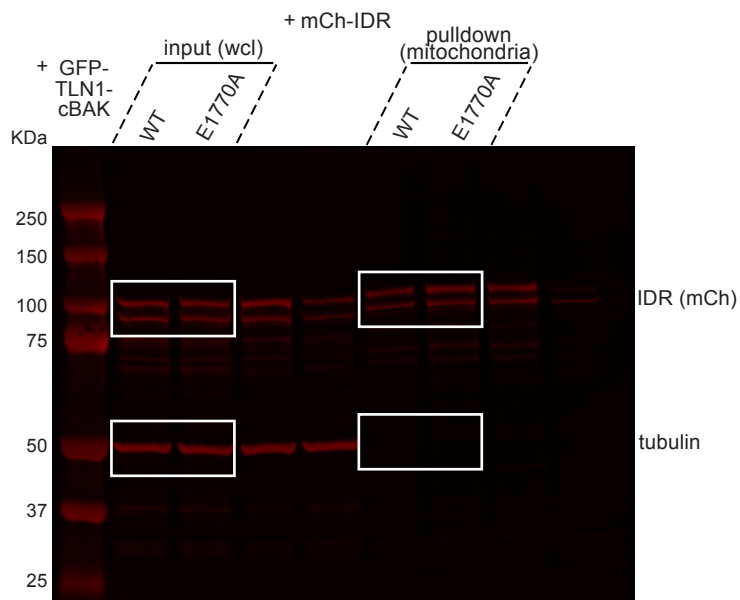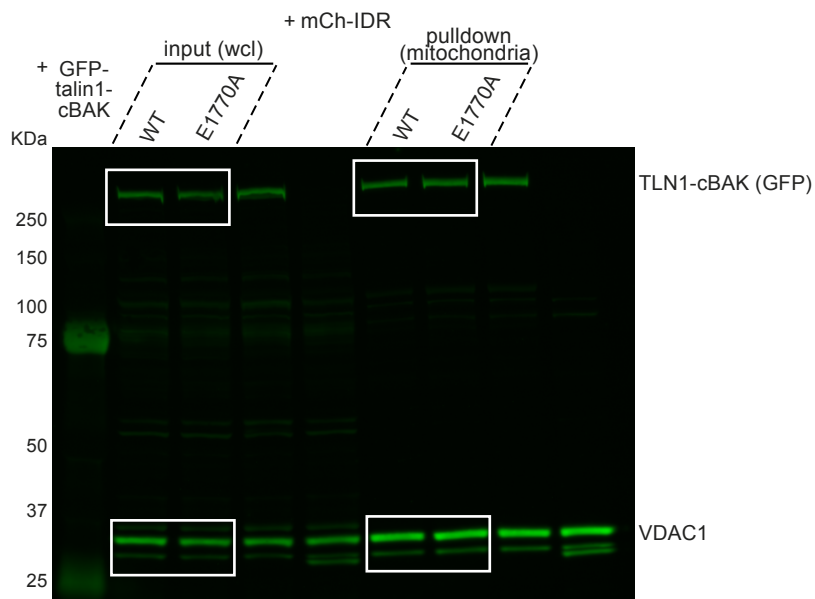

Supplement: SourceData F7 — is the source file for Fig. 7. [file jcb_202503155_sourcedataf7.pdf]

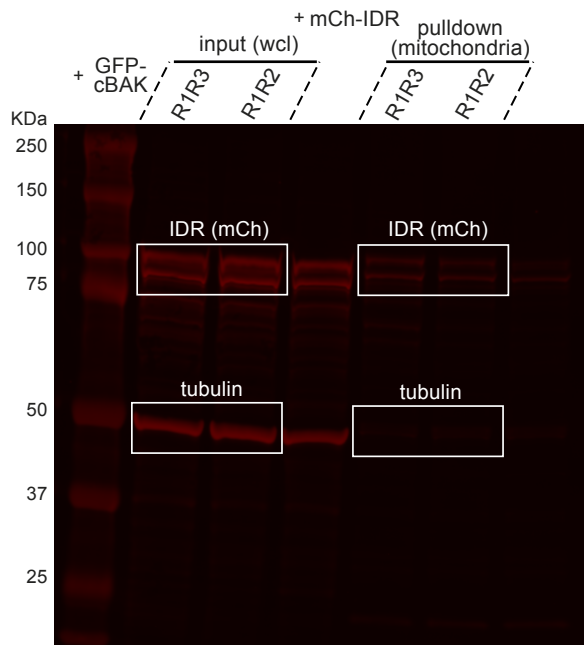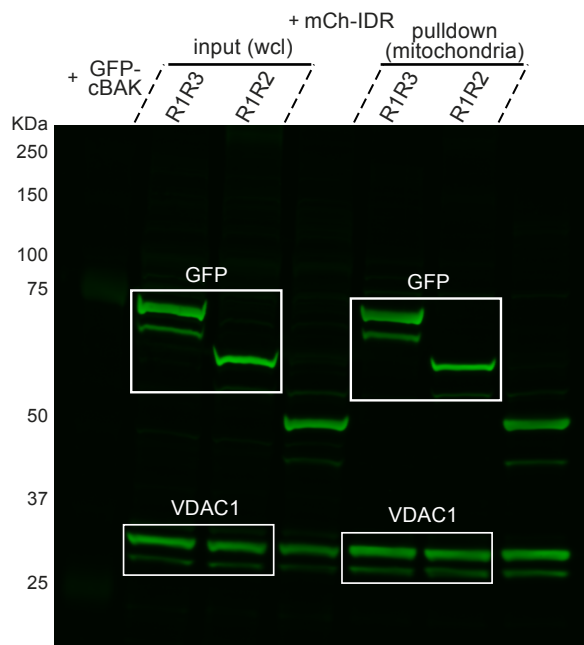

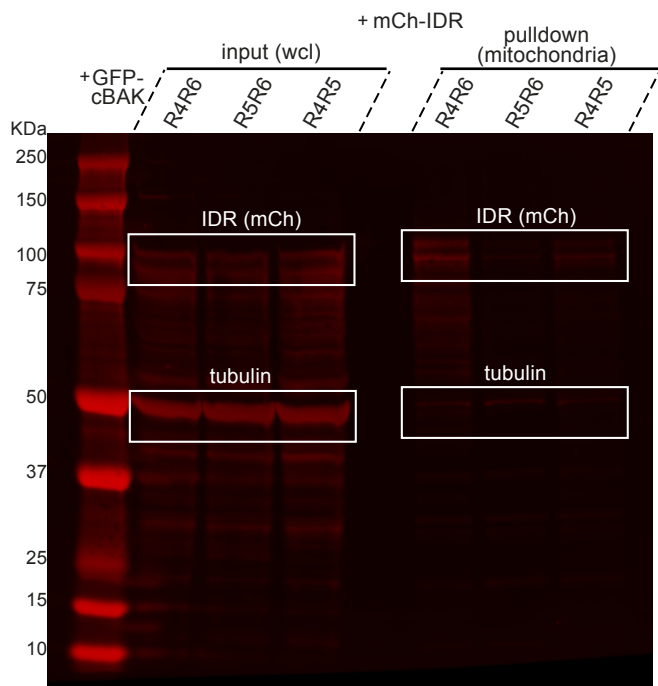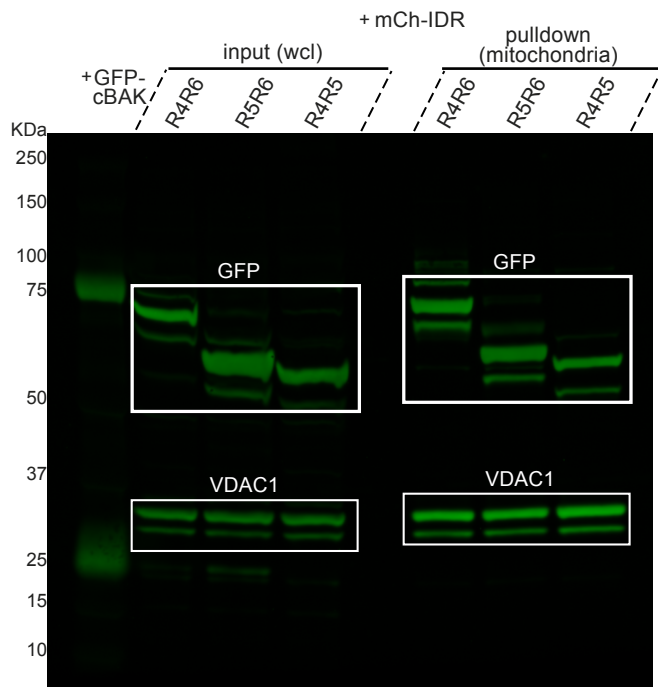

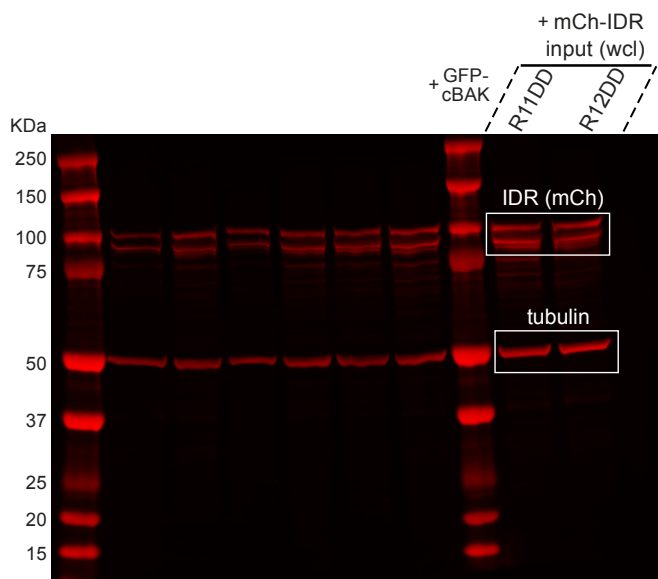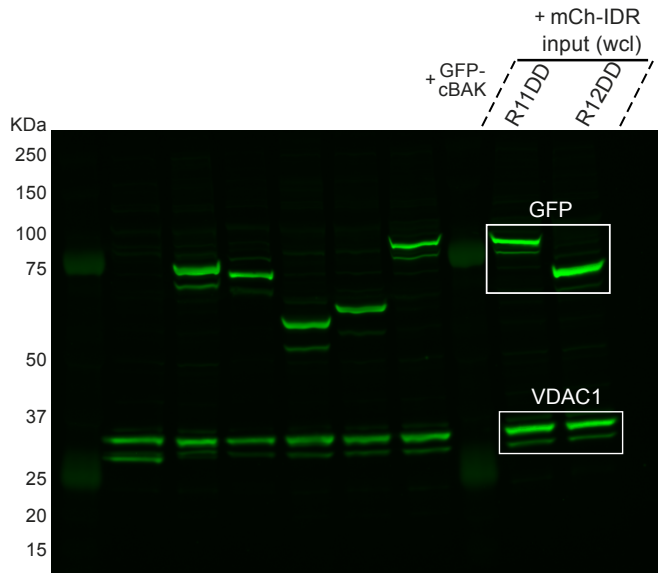

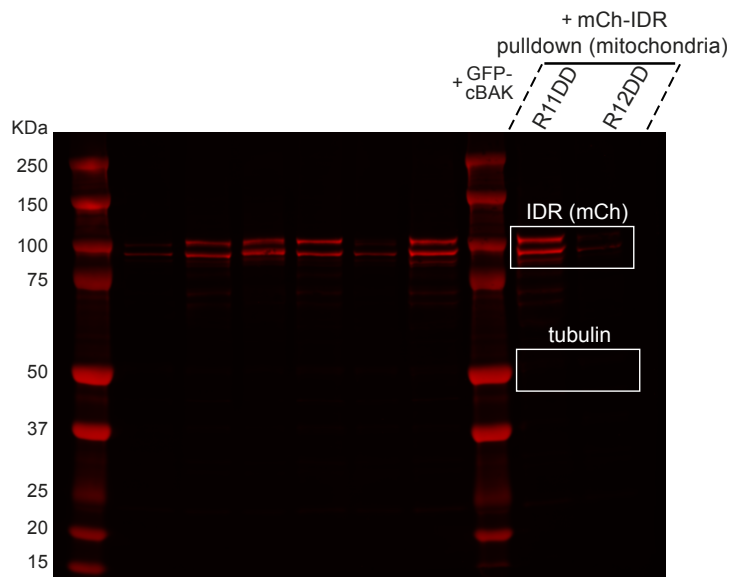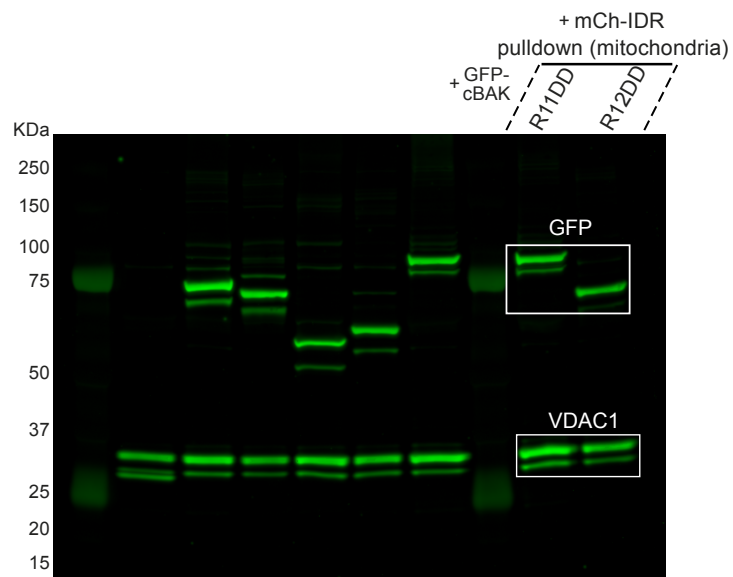

Supplement: SourceData FS1 — is the source file for Fig. S1. [file jcb_202503155_sourcedatafs1.pdf]
